# Association between frailty and clinical outcomes in patients undergoing craniotomy—systematic review and meta-analysis of observational studies

**DOI:** 10.1186/s13643-024-02479-3

**Published:** 2024-02-23

**Authors:** Ana Licina, Andrew Silvers, Christopher Thien

**Affiliations:** 1 Victorian Heart Hospital, Melbourne, Victoria Australia; 2 Monash Health, Clayton, Australia; 3 St. Vincent’s Hospital, Sydney, Australia

**Keywords:** Craniotomy, Frailty, Clavien–Dindo Classification, Neurosurgery

## Abstract

**Background:**

Frailty in patients undergoing craniotomy may affect perioperative outcomes. There have been a number of studies published in this field; however, evidence is yet to be summarized in a quantitative review format. We conducted a systematic review and meta-analysis to examine the effects of frailty on perioperative outcomes in patients undergoing craniotomy surgery.

**Methods:**

Our eligibility criteria included adult patients undergoing open cranial surgery. We searched MEDLINE via Ovid SP, EMBASE via Ovid SP*,* Cochrane Library, and grey literature. We included retrospective and prospective observational studies. Our primary outcome was a composite of complications as per the Clavien–Dindo classification system. We utilized a random-effects model of meta-analysis. We conducted three preplanned subgroup analyses: patients undergoing cranial surgery for tumor surgery only, patients undergoing non-tumor surgery, and patients older than 65 undergoing cranial surgery. We explored sources of heterogeneity through a sensitivity analysis and post hoc analysis.

**Results:**

In this review of 63,159 patients, the pooled prevalence of frailty was 46%. The odds ratio of any Clavien–Dindo grade 1–4 complication developing in frail patients compared to non-frail patients was 2.01 [1.90–2.14], with no identifiable heterogeneity and a moderate level of evidence. As per GradePro evidence grading methods, there was low-quality evidence for patients being discharged to a location other than home, length of stay, and increased mortality in frail patients.

**Conclusion:**

Increased frailty was associated with increased odds of any Clavien–Dindo 1-4 complication. Frailty measurements may be used as an integral component of risk-assessment strategies to improve the quality and value of neurosurgical care for patients undergoing craniotomy surgery.

**Ethics and dissemination:**

Formal ethical approval is not needed, as primary data were not collected.

**Systematic review registration:**

PROSPERO identification number: https://www.crd.york.ac.uk/prospero/display_record.php?RecordID=405240

**Supplementary Information:**

The online version contains supplementary material available at 10.1186/s13643-024-02479-3.

## Background

Craniotomy is used to treat a number of intracranial conditions, including brain tumors, arteriovenous malformations, arterial aneurysms, acute and chronic hemorrhage, and a number of congenital conditions [1]. Planning for surgery requires an informed risk discussion, including the benefits of surgery and the likelihood of complications. In patients undergoing craniotomy, there are limited risk assessment tools to assist clinicians and patients with decision-making. There is a clinical need for a standardized, validated, preoperative risk assessment tool to provide informed consent and aid in patients’ decision-making. As such, pre-operative evaluation inclusive of frailty should be considered part of the process of informed consent for medical intervention. This active discussion and quantifiable risk provision would pertain to outcomes should frail patients decide to proceed with surgical management. Frailty is now an established risk assessment tool in a multitude of surgical specialties [2–5]. Frailty was defined as a clinical syndrome in which three or more of the following criteria were present: unintentional weight loss (10 pounds in the past year), self-reported exhaustion, weakness (grip strength), slow walking speed, and low physical activity [6]. Measurement of frailty indices and correlation with the likelihood of peri-operative complications may assist clinicians and patients with decision-making during the surgical process [5].

Two commonly recognized conceptual frameworks for frailty are the phenotypic framework and the deficit accumulation model [7]. The phenotype framework is based on a group of physical signs and symptoms, including physical characteristics (weight loss, weakness, exhaustion, slowness, and low activity), and is associated with reduced levels of energy [8]. Examples of instruments using the phenotype framework include the Physical Frailty Phenotype, Frailty/Vigor Assessment, and Clinical Frailty Scale [9]. The deficit accumulation model is a multiple aggregate domain model that relies on the number rather than the nature of health problems [10, 11]. A correlation exists between the two constructs of frailty, with studies demonstrating overlap of the two classifications of frailty measurement instruments. Studies have demonstrated both construct and content validity among frailty instruments [7, 10–12]. Of these, the modified Frailty Index-5 derived from the modified Frailty Index-11 and John Hopkins Frailty Instrument, among others, are frequently used. Other systematic reviews and meta-analyses have included measurements of different instruments of frailty to allow for pooled effect estimates [3, 13, 14].

In the published individual studies on the association of frailty with cranial surgery, patients who are considered frail experience higher rates of complications, operative mortality, and hospital length of stay [15–17]. While frailty is a spectrum, in this study we sought to differentiate frail from non-frail patients. There is a clinical need to summarize the pooled data on the impact of frailty in cranial surgery in a quantitative manner. This would improve the incorporation of frailty into risk assessment modeling in cranial surgery. We sought to address this unmet clinical need by conducting a systematic review and meta-analysis of the association of frailty with perioperative outcomes, including the overall rate of complications within 30 days, perioperative mortality and discharge disposition within 30 days, and length of stay in adult patients undergoing open cranial surgery (craniotomy).

## Methods including statistical analysis

### Protocol and registration

The protocol for this review was registered prospectively within the International Prospective Register of Systematic Reviews CRD42023405240. This systematic review was conducted in accordance with the methodology for meta-analysis of observational study design [18]. The present review is being reported in accordance with the Preferred Reporting Items for Systematic Reviews and Meta-Analyses (PRISMA) Statement [19].

### Eligibility criteria

We included prospective and retrospective observational studies that reported frailty in patients undergoing open cranial surgery. Patients who were deemed to be not frail were considered the observational control group. We included all patients older than 18 who had undergone a craniotomy. We included patients undergoing craniotomy procedures for any of the following pathologies or locations: brain tumor, benign or malignant, aneurysm surgery, intracranial hemorrhage, or anterior or posterior fossa surgery. We excluded patients undergoing minimally invasive surgery and burr hole surgery.

Previous literature has noted that systematic reviews and meta-analyses should include all available evidence to avoid selection bias and to increase the power of analyses of primary effects by differences in patients and interventions. We considered any tool to measure frailty as eligible. We defined frailty as per the study’s definition as being frail and non-frail. The absence of frailty was identified by studies as the absence of frailty qualifiers [20]. We pooled the non-frail or lowest frailty score group as the reference group. We included studies published in the English language. We did not introduce a time limit in our eligibility criteria. Studies were excluded if they were not an original research contribution. Studies were also excluded if they were single-arm studies only, systematic reviews, conference presentations, or letters to the editor.

### Information sources and search strategy

We searched MEDLINE via Ovid SP; EMBASE via Ovid SP; and the Cochrane Library (Cochrane Database of Systematic Reviews and CENTRAL). We searched the grey literature [21–23]. We completed our searches in March 2023. For the search strategy, we combined keyword and subject heading combinations in the predetermined databases (Supplementary file-Search Strategy) [18].

References of included studies were searched for any other potentially eligible studies for inclusion.

### Data management

Study information was stored and managed using Endnote X9 [24]. Two reviewers independently screened titles and abstracts for inclusion. When there was disagreement, this was reviewed and determined by the third author. Data were extracted by a single reviewer. Relevant outcome data were collected as presented by the studies. Extracted data were confirmed by a second reviewer. We included articles for full-text review unless both reviewers deemed them irrelevant. Data were extracted using a prespecified extraction form. The results of the data search have been presented in a PRISMA flow diagram. Authors of primary publications were contacted for data clarifications or missing outcome data.

### Outcomes and prioritization

We used the Clavien–Dindo model to grade and define perioperative complications [25, 26]. This classification system has been identified to be reliable and reproducible in the surgical literature (Table 1).

**Table 1:** Classification of surgical complications

*Grade definition*	
Grade I	Any deviation from the normal postoperative course without the need for pharmacological treatment or surgical, endoscopic, and radiological interventions Allowed therapeutic regimens are: drugs as antiemetics, antipyretics, analgetics, diuretics, electrolytes, and physiotherapy. This grade also includes wound infections opened at the bedside.
Grade II	Requiring pharmacological treatment with drugs other than such allowed for grade I complications.
	Blood transfusions and total parenteral nutrition are also included.
Grade III	Requiring surgical, endoscopic, or radiological intervention.
Grade IIIa	Intervention is not under general anesthesia.
Grade IIIb	Intervention under general anesthesia.
Grade IV	Life-threatening complication (including CNS complications)^a^ requiring IC/ICU management.
Grade IVa	Single organ dysfunction (including dialysis).
Grade IVb	Multiorgan dysfunction.
Grade V	Death of a patient.

*CNS* Central nervous system, *IC* Intermediate care, *ICU* Intensive care unit

^a^Brain hemorrhage, ischemic stroke, subarachnoid bleeding, but excluding transient ischemic attacks

Our primary outcome was defined as the overall number of complications experienced by patients within 30 days. We defined the complications according to the Clavien–Dindo classification system. We considered any systemic, neurological, or regional complication as included in the overall complication number (Clavien–Dindo 1–4). This has been defined as any composite score of cardiovascular, pulmonary, neurological, thromboembolic, or infectious complications. We used the weighted composite model to calculate this outcome [27]. Our secondary outcomes consisted of length of hospital stay, frailty and discharge disposition, hospital readmission within 30 days, and mortality within 30 days.

### Measures of association

For dichotomous outcomes, we obtained an odds ratio (OR) from the group with the exposure (frail group) and control (non-frail) group event rates. Dichotomous outcomes included complications in the frail versus non-frail group as defined by the primary outcome, frailty and discharge disposition, hospital re-admission within 30 days, and mortality within 30 days. For continuous data, we obtained the mean difference (MD) with the associated standardized mean difference (SMD). Continuous outcomes included length of hospital stay.

### Risk of bias in individual studies

The risk of bias in the included studies was assessed using the Cochrane “Risk of Bias” tool in non-randomized studies [28]. We included a “risk of bias” table. We generated a “risk of bias summary” [29].

### Data synthesis—planned summary measures and methods of handling and combining data

As different measurement tools were used to assess common outcomes, the results were pooled using random effects meta-analyses to calculate summary estimates using Revman software. We used Review Manager 5.3 Software for statistical analysis. We used the inverse variance weighting summary of continuous outcomes and Mantel–Haenszel methods for dichotomous outcomes [30]. We generated odds ratios for binary outcomes and standardized mean differences for continuous outcomes. The outcomes were presented with 95% confidence intervals. We used the data presented by the studies, as clinical frailty assessment is typically employed as a risk stratification tool, as opposed to as a part of a multivariable risk model.

We performed a sensitivity analysis, where we excluded studies at the highest risk of bias. As part of sensitivity analysis, we planned to analyze the effects of methodology—primary outcome only in the patients undergoing cranial surgery in studies with prospective methodology only. Where we identified significant heterogeneity, we planned to conduct separate subgroup analyses to explore potential causes of heterogeneity and account for inherent bias due to selection, classification, and confounders among the different studies. For all tests, significance was defined as *p* < 0.05.

We reported statistical heterogeneity using the Chi^2^ statistic and the *I*^2^ statistic. Both were calculated for each of the outcomes listed above. Statistical heterogeneity was declared if the Chi^2^ statistic had *P* < 0.1. We evaluated the importance of *I*^2^ depending on the magnitude and direction of effects as well as the strength of evidence for heterogeneity [18]. We determined heterogeneity as not important for *I*^2^ of 0 to 40%, as moderate for *I*^2^ of 30 to 60%, as substantial for *I*^2^ of 50 to 90%, and as considerable for *I*^2^ of 75 to 100%. Publication bias was tested by funnel plots (Metafunnel in STATA) using Egger’s test [31].

### Confidence in cumulative evidence—summary of findings’ tables and Grading of Recommendations, Assessment, Development and Evaluation (GRADE)

The quality of the evidence needs to be appraised to the extent to which one can be confident that the estimates of effect reflect the items assessed. We used the GRADE classification system to rate the quality of the body of evidence across individual outcomes in observational studies [32, 33]. We generated the “Summary of findings” table using GRADEpro software for observational studies. We constructed a “Summary of findings” table for the primary outcome. The “Summary of findings” table was supported by the Evidence Profile Table [34]. There are five areas evaluated within the body of evidence: within-study risk of bias (methodological quality), indirectness, heterogeneity of data (inconsistency), imprecision of effect estimates, and risk of publication bias.

### Subgroup analysis

As preplanned in the protocol, three subgroup analyses were conducted based on clinical and methodological assumptions. We planned the following subgroup analysis:

1. Patients undergoing cranial surgery for tumor surgery only.
2. Patients undergoing cranial surgery for non-tumor surgery only.
3. Patients older than 65 years undergoing cranial surgery.

## Results

### Results of the search

**Fig. 1:**
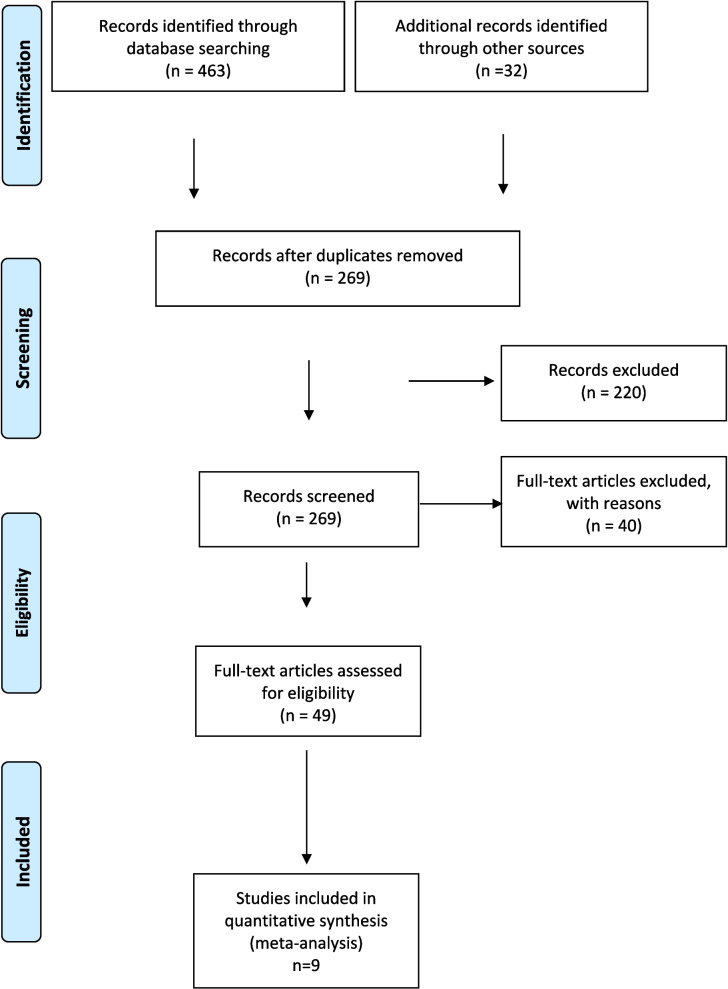
PRISMA Diagram of eligible studies

We identified a total of 463 articles. After excluding duplicates, 23 studies were included for full-text review. We included nine studies [5, 16, 17, 35–40]. The results of the literature search process are graphically presented in a PRISMA flow chart (Fig. 1 Prisma diagram).

### Summary of included studies

A total of nine studies were eligible for inclusion. Eight studies were retrospective, and one study was prospective. The single prospective study was performed by Harland et al. [37]. Retrospective studies analyzed data from surgical databases, including the National Surgical Quality Improvement Program (NSQIP). In prospective studies, frailty was calculated prior to patients undergoing surgery [37]. Two studies included patients over 65 years of age only, for a total of 13,585 patients [16, 35]. Seven studies analyzed tumor-only cases, for a total of 45,544 patients. Two other studies included other pathologies in their review, including acute cerebral hemorrhage and posterior fossa surgery [36, 38]. Please see Table 2 for the summary of included studies.

**Table 2:** Summary characteristics of included studies

Study	Condition studied	Study design	Number of participants	Participant characteristics	Frailty scale used and percentage	Outcomes	Results
Cloney et al 2015 [35]	frailty in patients with glioblastoma	Retrospective cohort study in elective surgery	243 patients	Age > 65	MFI 11 81% frail	-Association between mFI score and the decision to forego surgical resection -rate of postoperative complications, -Length of hospital stay, -Overall mortality;	-Frailer patients were less likely to undergo surgical resection, as opposed to a biopsy, had longer hospital stays, an increased overall risk of complications, and decreased overall survival;
Harland et al. 2020 [37]	frailty in patients undergoing tumour surgery	Prospective cohort study in elective surgery	260 patients	Age > 18	John Hopkins Frailty Instrument 25% frail	-Postoperative complications within 30 days of surgery, including mortality; -new neurologic deficit; -LOS; -discharge to a skilled nursing facility, acute rehabilitation facility, or hospice at 30 days when previously independent	-Preoperative frailty was associated with an increased risk for discharge to a location other than home, postoperative complications;
Henry et al. 2021 [38]	skull base procedures National Surgical Quality Improvement Program (NSQIP) database from 2005 to 2018	Retrospective cohort study—trauma cases were excluded	17912	Age > 18	5 Factor Modified Frailty Index 45% frail	Primary outcome variables included rates of overall complications and life-threatening complications within the 30-day postoperative period.	-Independent predictor of overall complications life-threatening complications, and mortality.
Huq et al. 2021 [15]	Frailty in brain tumor patients	Single-center retrospective cohort study primary brain surgery	1692	Age > 18	5 Factor Modified Frailty Index 57% frail	Total length of stay (LOS), intensive care unit (ICU) LOS, complications, charges, and 30-d readmissions)	-Mean intensive care unit (ICU) and total LOS were 1.69 and 5.24 days respectively. Mean pulmonary embolism (PE)/deep vein thrombosis (DVT), physiological/metabolic derangement, respiratory failure, and sepsis rates were 7.2%, 1.1%, 1.6%, and 1.7%, respectively. Mean total charges were $42,331;
Imaoka et al. 2018 [36]	Frailty in patients undergoing treatment for spontaneous cerebral hemorrhage	Single-center retrospective cohort study—no trauma cases	156	Age > 18	11 Factor Modified Frailty Index 75% frail	Outcome measures included an unfavorable outcome (modified Rankin Scale score of 4–6) or mortality at 6–8 months after hemorrhage.	-Higher mFI was significantly associated with an unfavorable outcome (*p* value = 0.004) and mortality
Shahrestani et al. 2020 [16]	Frailty in patients undergoing primary tumor surgery	Primary CNS neoplasm between 2010 and 2017 by using the Nationwide Readmission Database	13342	Age > 65	JHACG 50% frail	-Demographics and frailty were queried at primary admission, and readmissions were analyzed at 30-, 90-, and 180-day intervals; complications of interest included infection, anemia, infarction, kidney injury, CSF leak, urinary tract infection, and mortality;	-Mortality was increased compared to non-frail geriatric patients receiving the same procedure; frail patients had a significantly increased inpatient length of stay (*p* < 0.0001) and all-payer hospital cost (*p* < 0.0001) compared to non-frail patients at the time of primary admission.
Theriault 2020 [40]	Primary tumor surgery	Single-center retrospective cohort study of patients who underwent intracranial meningioma resection	76 patients	Age> 18	5 Factor Modified Frailty Index; 55% frail	LOS (length of stay), discharge location, readmission rates, and reoperation rates;	-Increased hospital LOS (*p* = 0.0218), increased reoperation rate (*p* = 0.029), and discharge to a higher level of care;
Sastry et al. 2020 [39]	Primary tumor surgery	Retrospective cohort 2012–2018 ACS-NSQIP participant use file	20,333	Age> 18	5 Factor Modified Frailty Index 41% frail	incidence of major postoperative complications, discharge destination other than home, 30-day readmission, and 30-day mortality after elective craniotomy for brain tumor resection.	- Both low and medium-high frailty were associated with increased adjusted odds ratio of major complications, discharge destination other than home, and 30-day mortality;
Youngerman et al. 2017 [17]	Primary tumor surgery	NSQIP 2008–2012	9149	Age> 18	11 Factor Modified Frailty Index 49% frail	30-day mortality, 30-day severe medical complications, 30-day severe neurologic complications, 30-day any complication, extended length of stay (LOS), and unfavorable disposition	-mFI was associated with stepwise increases in the rates of mortality, severe medical complications, prolonged length of stay, and unfavorable discharge;

Of the 63,159 patients included, 29085 (46%) were classified as frail. The pooled mean age (SD) was 60 (13.5). Individual comprehensive study characteristics have been presented in the additional file (Supplementary file-Study characteristics). Across all studies, frailty was measured using 4 different instruments. The characteristics of the frailty instruments used are summarized in Table 3.

- 11 factor modified Frailty Index (mFI 11);
- 5 factor modified Frailty Index (mFI 5);
- John Hopkins Frailty Instrument;
- Johns Hopkins Adjusted Clinical Groups (JHACG) frailty-defining diagnosis indicator;

**Table 3:** Frailty instruments used by eligible studies

Frailty instrument	Classification	Neurofrailty instrument description	Studies utilizing the index
11 Factor Modified Frailty Index (mFI-11)	Accumulating deficit model	This scale accounts for 11 variables, and 1 point is given for each variable present: Non-independent functional status History of diabetes mellitus History of chronic obstructive pulmonary disease History of congestive heart failure History of myocardial infarction History of percutaneous coronary intervention, cardiac surgery, or angina Hypertension requiring the use of medication Peripheral vascular disease or rest pain Impaired sensorium Transient ischemic attack or cerebrovascular accident without residual deficit Cerebrovascular accident with deficit	Cloney et al. [35] Imaoka et al. [36] Youngerman, et al. [17]
5 Variable Modified Frailty index (mFI-5)	Accumulating deficit model	The mFI-5 is calculated using the following variables: -non-independent functional status, -Diabetes mellitus, -Chronic obstructive pulmonary disease or current pneumonia, -Congestive heart failure, and -Hypertension requiring medication. Non-independent functional status is defined by the NSQIP database as requiring assistance for any activities of daily living, including feeding, dressing, bathing, and mobility. Each factor contributes one point for an mFI-5 score between 0 and 5, with increasing scores implying increasing frailty.	Henry et al. [38] Huq et al. [15] Therioult et al. [40] Sastry et al. [39]
John Hopkins Frailty Instrument	Phenotype deficit model	This phenotype model of frailty includes the 5 components of the HFS: shrinking, weakness, exhaustion, low activity, and slowed walking speed	Harland et al. [37]
Johns Hopkins Adjusted Clinical Groups (JHACG) frailty-defining diagnosis indicator	Phenotype deficit model	Johns Hopkins Adjusted Clinical Groups frailty-defining diagnosis indicator. The JHACG frailty-defining diagnosis indicator uses a set of 10 clinical clusters: malnutrition, dementia, vision impairment, decubitus ulcer, urine control, weight loss, fecal control, social support, difficulty walking, and history of a fall.	Shahrestani et al. [16]

Seven out of nine studies eligible for inclusion in this meta-analysis used the MFI 5 or the MFI 11 frailty instruments, for a total of 49,557 patients or 78.5% of the patient cohort included in the review.

### Primary outcome

**Fig. 2:**
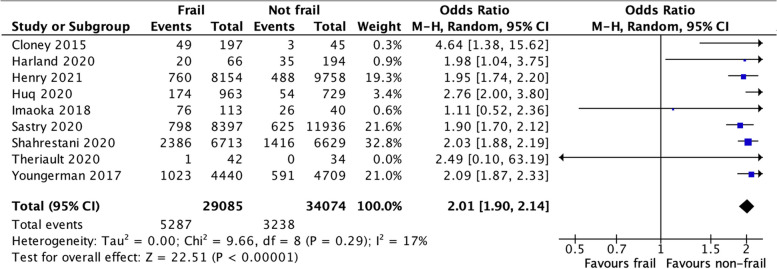
Clavien–Dindo complications in patients with frailty compared to the non-frail cohort

We assessed the clinical homogeneity of studies as suitable for meta-analysis. We noted no appreciable statistical heterogeneity for the primary outcome of Clavien–Dindo grade 1–4 complications in patients undergoing craniotomy. There were a total of 63159 patients suitable for analysis of the primary outcome. The odds of any complication developing in frail patients compared to non-frail patients was 2.01 (Fig. 2), with no identifiable statistical heterogeneity.

We used RevMan to generate a graphical representation of Egger’s test. Egger’s test for funnel plot symmetry was performed using Stata [16] for the total number of complications. *P* values for Egger’s test failed to reach statistical significance. We did not identify small study/publication bias (Supplementary data-Funnel plot assessment of publication bias).

### Secondary outcomes

The length of hospital stay was measured in 5 studies with a total of 15,856 patients***.*** The length of hospital stay was shorter in the non-frail group (0.75 [0.49, 1.01], *p* < 0.0001); however, this outcome was associated with considerable heterogeneity. In a total of five studies and 43,160 patients, frail patients were more likely to be discharged to a location other than home (OR 2.16 [1.77, 2.64], *p* < 0.0001), although this outcome was associated with substantial heterogeneity (*I*^2^ 68%). A total of 34,251 patients were included in the 30-day hospital readmission rates. The odds of readmission did not reach statistical significance (OR 1.25 [0.81, 1.93, *p* = 0.32). Mortality within 30 days was reported in three studies, with a total of 42,977 patients. Mortality was increased in the frail cohort (OR 2.67 [1.53, 4.68], *p* < 0.00001); however, this outcome was associated with substantial heterogeneity (*I*^2^ = 91%) (Supplementary data-Secondary outcomes).

### Sensitivity analysis

To conduct sensitivity analysis, we excluded a single prospective study. The robustness of summary statistics was maintained across the primary outcome (OR 2.02 [1.89, 2.16]), with low heterogeneity. However, retrospective studies accounted for 99% of the patient population in this study.

We excluded studies deemed at higher risk of bias [16]. The robustness of summary statistics was maintained across the primary outcome (OR 2.02 [1.84, 2.21]), with low heterogeneity (Supplementary data-Sensitivity analysis).

### Subgroup analysis for primary outcome

We conducted three subgroup analyses as planned in the protocol. We conducted the analysis for the overall odds of complications in patients undergoing tumor surgery only. The odds of complications were similar to the primary analysis with no appreciable statistical heterogeneity, 2.0 [1.91, 2.17], *p* < 0.0001.

We conducted a subgroup analysis of patients undergoing non-tumor surgery. Two studies were included in this subgroup. In patients undergoing non-tumor surgery, the odds of frail patients having any complication were 1.67 [1.02, 2.75] *p* = 0.04, with moderate heterogeneity (Supplementary data-Subgroup analysis).

We conducted a subgroup analysis of patients older than 65 years undergoing any type of surgery. Only Cloney et al. and Shahrestani et al. included patients over 65 years of age, studies that included patients who underwent tumor surgery [16, 35]. In a subgroup analysis of these two studies only, we noted a wider confidence interval with moderate heterogeneity (OR 2.43 [1.24, 4.76], *p* = 0.009).

### Post hoc analysis

#### Deficit accumulation models compared to the phenotype frailty model

**Fig. 3:**
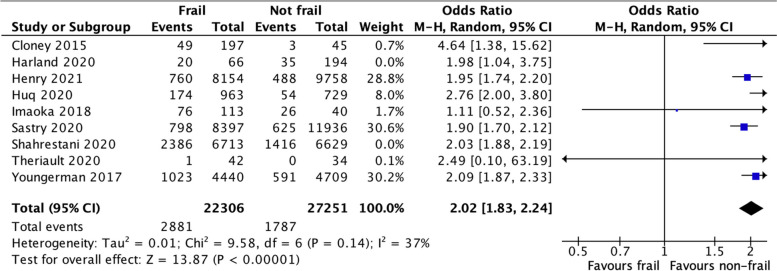
Frailty using the deficit accumulation model only

In our post hoc analysis, we identified studies that used the cumulative deficit model of frailty only. Seven studies with a total of 49,557 patients used cumulative frailty measurement instruments. The odds of any complication in the studies using the deficit accumulation model frailty instruments (mFI-5 and mFI-11) were 2.02 [1.83, 2.24] *p* < 0.0001, with moderate heterogeneity (Fig. 3).

Two studies used the phenotype construct of frailty to determine frailty in a total of 13602 patients [16]. The odds of having a complication were 2.03 [1.88, 2.19], *p* < 0.0001. This group was dominated by a single retrospective large study by Shahrestani et al. [16]. Although there were a small number of studies measuring the phenotype construct compared with the deficit accumulation model, the odds of any complication in the two frail groups were similar (Supplementary data-Post-hoc analysis).

#### Likelihood of complications in the spectrum of frailty

**Fig. 4:**
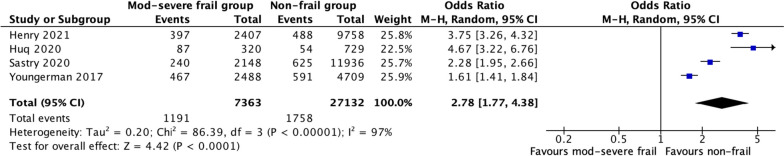
Complications in the moderately to severely frail patients

As frailty exists on a spectrum, in our post-hoc analysis we analyzed the quantifiable risk of complications in patients with the greatest level of frailty. A limited number of studies presented this data. We performed a meta-analysis of the likelihood of complications in the patients deemed moderately to highly severe, in comparison to the patients with no frailty. We identified that this group of patients had high odds of overall complications 2.78 [1.77, 4.38], albeit with high statistical heterogeneity (Fig. 4).

Studies excluded patients undergoing emergency/trauma surgery and as such we were unable to perform a post-hoc analysis on this group.

#### Primary outcome in smaller non-database studies

We tested the robustness of our findings by excluding large database studies. We performed this post-hoc analysis in order to evaluate the internal validity of findings, without the larger studies included. There were a total of five studies with 2423 patients. The odds ratio of complications was similar to that of the primary outcome (2.22 [1.46, 3.37], *p* = 0.0002), with low heterogeneity.

### Risk of bias of included studies

“Risk of bias results in the seven domains applicable to observational studies. Seven domains applicable in observational studies include, together with example criteria provided: D1: Bias due to confounding, for example, did the authors control for baseline factors?; D2: Bias arising from the measurement of the exposure, does the measurement of exposure characterize the metric of interest?; D3: Bias in the selection of participants into the study (or into the analysis), for example, did the patients’ follow-up commence at the start of the study?; D4: Bias due to post-exposure intervention, were there post-exposure interventions that influenced the study findings?; D5: Bias due to missing data, what missing data were identified? D6: Bias arising from the measurement of the outcome, how was the outcome measured?; D7: Bias in selection of the reported result, were results reported in line with available study protocols. For full details please see the ROBINS-E tool [41]”.

**Fig. 5:**
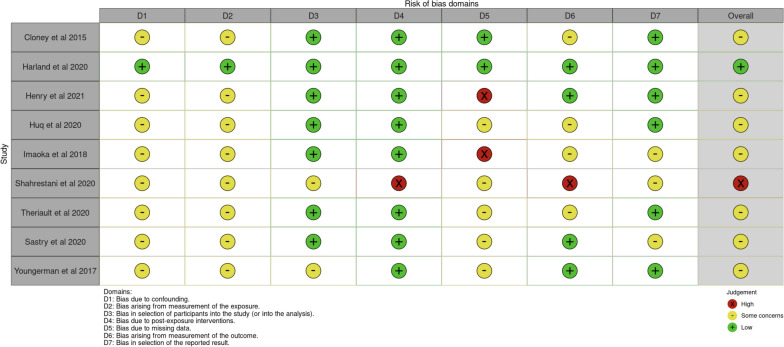
Risk of bias in individual domains

**Fig. 6:**
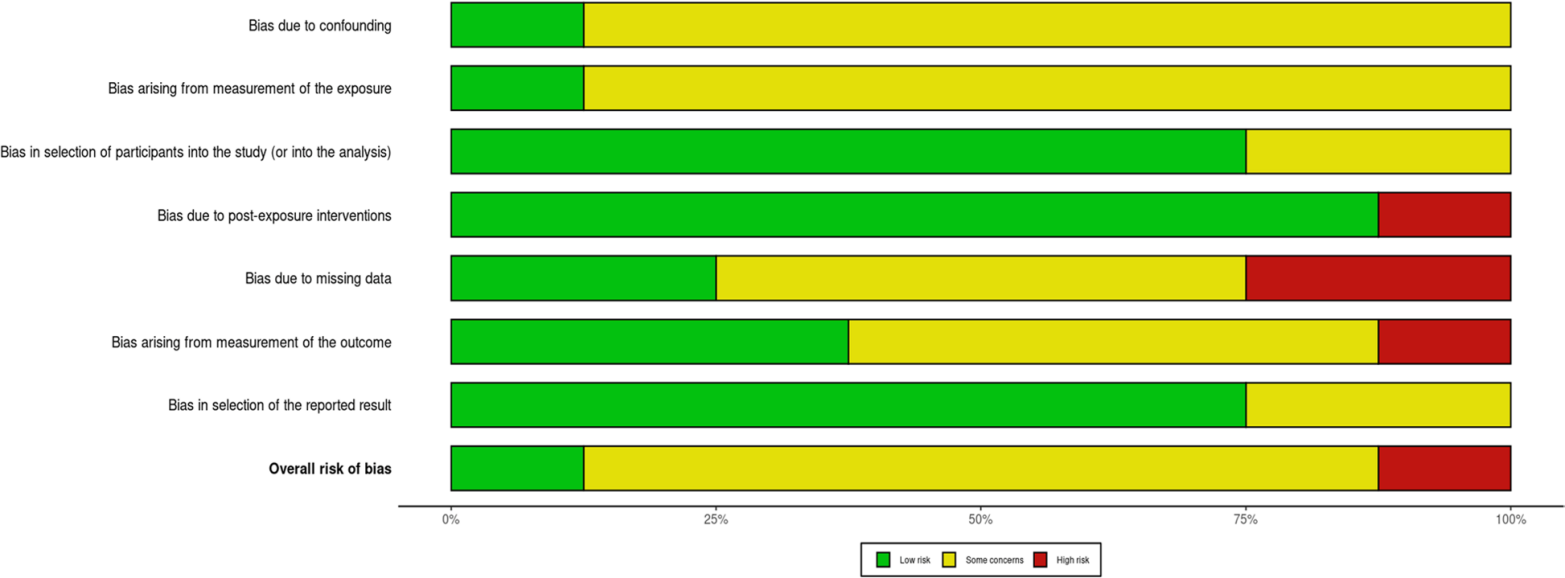
Risk of bias graph across domains

Domain 3, with bias in the selection of participants in the study, had low concerns. Most studies used appropriate criteria and identified eligible cases. We identified low bias in domain 7 and bias in the selection of reported results. Overall, studies were dominated by prospective nature, and therefore, there were some concerns with bias estimates in most studies (Figs. 5 and 6).

### Confidence in cumulative evidence—summary of findings’ tables and GRADE

We summarized the quality of evidence across primary and secondary outcomes in evidence profile tables. We assessed the evidence across the primary outcome of the odds of Clavien–Dindo 1–4 complications as a serious risk of bias due to the retrospective nature of the studies. For the primary outcome, we identified moderate-quality evidence, with low inconsistency and low imprecision [42]. We repeated the process of grading the evidence across the secondary outcomes. We identified low-quality evidence for discharge to a location other than home and very low-quality evidence for the outcomes of length of stay and mortality.

GRADEpro software was utilized to generate the “Evidence Profile Tables” and “Summary of Findings” tables (Supplementary data-Evidence Profile Tables and Summary of Findings data).

## Discussion

In this meta-analysis of observational studies in patients undergoing craniotomy, we identified that patients with frailty have increased odds of Clavien–Dindo 1–4 complications. Evidence for this outcome was of moderate quality with very low statistical heterogeneity. This outcome maintained statistical robustness across sensitivity analysis as well as post hoc analysis according to the frailty model (phenotype or deficit accumulation model) used. Frailty was associated with adverse secondary clinical outcomes, albeit with associated statistical heterogeneity. Frail patients were twice as likely to be discharged to a location other than home; however, evidence for this was low due to marked heterogeneity. Length of stay and mortality were higher in the frail group; however, these outcomes were associated with marked heterogeneity and very low quality of evidence.

This is the first meta-analysis in patients undergoing open cranial surgery, indicating increased odds of complications in frail patients. We identified higher odds of any complication, as classified according to the Clavien–Dindo 1–4 groups, in frail patients undergoing open cranial surgery. This was a robust outcome with very persistent low statistical heterogeneity across sensitivity analysis and subgroup analysis. The results of this meta-analysis allow for quantification of the greater likelihood of complications in frail patients, thereby facilitating surgical decision-making and patient perioperative pathways. Although there are no other comparative meta-analysis data, the likelihood of complications was increased in a recently published meta-analysis of patients undergoing cardiac surgery [3].

Frail patients had more adverse outcomes than the non-frail cohort; however, these outcomes were associated with significant heterogeneity. Frail patients were more likely to be discharged to a location other than home. Quality of life outcomes, such as discharge to a non-home location (NHD), are important to patients and should be considered part of the process of informed consent for medical intervention. NHD has been shown to be associated with decreased overall survival and significant healthcare and social costs [43].

However, in our study for the non-home discharge outcome, the quality of evidence was downgraded due to heterogeneity impacting the inconsistency criterion. Further studies focusing on clinical homogeneity are needed. The rates of readmission failed to reach statistical significance. Our research findings show parallels with large database studies in related fields [44, 45]. Our findings are in line with the quantitative analysis of other surgical and intensive care groups [13]. Greater statistical heterogeneity may be due to the clinical in-group differences between frail patients in patients undergoing open cranial surgery. A more sophisticated approach to studying the outcomes in frail patients undergoing craniotomy would involve stratification according to the level of frailty in the original studies.

The pooled prevalence of frailty in this study was 48%, ranging from 21 to 85%. A diverse range of frailty may be contributed to by a diverse range of cranial pathology; therefore, the patient population needs to undergo surgery. Our study has a higher pooled prevalence of frailty compared to recent meta-analyses in other fields [13]. The reasons for this are unclear. The development of cranial pathology may have a role in increasing preoperative frailty at the time of measurement.

We did not identify marked heterogeneity in the measurement instruments used. MFI-5 is a tested derivative of MFI-11, and the majority of studies used one of these instruments (7 studies in total incorporating 49,557 patients). With regard to large retrospective database studies, the mFI-5 is user-friendly. The two studies that used the phenotype frailty model utilized the John Hopkins Frailty Instrument or the related John Hopkins Adjusted Clinical Group’s frailty-defining diagnosis indicator. In the post hoc analysis, we identified the odds ratios of a primary outcome occurring as similar between the two groups, with no appreciable significant differences. Although most studies have utilized deficit accumulation model instruments, it is unclear whether deficit accumulation models or phenotype models are better instruments for frailty.

Consensus on a unified measurement instrument of frailty would eliminate instrument-related heterogeneity and streamline the research processes. However, there may be some benefits lost from multitool validation. Studies have demonstrated that the 5-factor modified frailty index (mFI-5) and the 11-factor modified frailty index (mFI-11) are equally effective in predicting adverse outcomes in the American College of Surgeons National Surgical Quality Improvement Program database [46]. They have both been identified to be equally predictive of postoperative complications [47]. The MFI-5 index has been deemed credible for future use to study frailty within NSQIP, within other databases, and for clinical assessment and use [46, 48, 49]. Khallafah et al. specifically demonstrated the validity and responsiveness across the mFI-5, mFI-11, and Charleston comorbidity index in the neurosurgical field [50].

Based on the findings of this meta-analysis, we postulate that frailty measurements could be used as an integral component of strategies to improve the quality and value of neurosurgical care for patients undergoing craniotomy surgery. Although there is a component of delivering timely care to patients undergoing cranial surgery, perioperative pathways can be streamlined to facilitate patient care. Multidisciplinary decision-making can be instituted to assess patient deficits and risks and formulate goals of care. Prehabilitation in patients facing urgent open cranial needs to be considered against the urgency of surgical management. There is evidence that prehabilitation as a component of a multidisciplinary approach improves patient-reported outcomes [51]. When it is feasible to do so in this patient population, prehabilitation may be utilized. Importantly, an increased odds ratio of complications in patients with frailty can be used as a part of informed surgical consent balanced with surgical benefits.

Limitations of this meta-analysis include the grouping of all categories of frail patients together in most of the index studies. There is a spectrum of frailty ranging from least frail to most frail. Our study was able to estimate a more comprehensive outcome assessment for all of the frail patients grouped together. More sophisticated observational research data are required prior to stepwise risk estimation according to patients in mild, moderate, or severe frailty grouping. “Further high-quality prospective studies which stratify the frailty groups independently of one another, are needed in order to provide a more accurate odds of complications in patients suffering from increasing frailty.”

It is likely that once these groups are stratified according to the level of frailty, a clearer picture with regard to association with complications would emerge. Our study encompassed all patients requiring cranial surgery. We performed planned subgroup and sensitivity analyses. However, we were unable to differentiate on the basis of tumor type. This study therefore included patients with glioblastoma, where the extent of resection is linked to prognosis [37]. This meta-analysis was only able to evaluate short-term outcomes for up to 30 days, with limited ability to review long-term outcomes as well as patient-reported outcomes (PROMs). Retrospective databases used by the studies (NSQIP) lack perioperative outcomes. Additional limitations of this meta-analysis include the retrospective design of seven studies. The use of retrospective chart reviewers and/or the NSQIP database may have introduced observational bias, as it relies on trained staff performing retrospective data collection. Data with regard to tumor histology, location, size, grade, and stage may be controlled for in a multivariable analysis; however, this level of detail is not available through a retrospective database.

A single study by Cloney et al. reported on the prevalence of frailty in patients with cranial pathology undergoing non-operative cranial management. Frailer patients (*P* 0.0002; odds ratio [OR], 0.15; 95% confidence interval [CI], 0.05–0.40) were significantly less likely to undergo surgical resection on multiple regression analysis. We were unable to source more comprehensive data on the prevalence of frailty in patients undergoing non-operative (conservative/palliative) cranial surgery. This may be valuable information to study in order to compare levels of frailty in patients undergoing craniotomy versus those who are not undergoing operative management.”

## Conclusion

These findings suggest that preoperative frailty assessments could assist in risk-stratifying patients undergoing open cranial surgery. Increased odds of complications in frail patients can assist decision-makers in perioperative pathway planning and with informed consent. Increased odds of complications were a statistically robust outcome across a number of subgroups and sensitivity analyses.

### Supplementary Information

**Additional file 1.** Ovid Medline Search Strategy.

**Additional file 2.** Characteristics of Included Studies.

**Additional file 3.** PRISMA_2020_checklist.

**Additional file 4.** Excluded studies with reasons.

**Additional file 5.** Supplementary Data.

## Data Availability

Data will be available beginning 9 months and ending 36 months after study result publication. Data will be shared with investigators whose proposed use of the data has been approved by an independent review committee (“learned intermediary”) identified for this purpose. Research proposals should be directed to analicina@hotmail.com. To gain access, data requestors will need to sign a data access agreement.
