# Supplementary material for: Association between frailty and clinical outcomes in patients undergoing craniotomy—systematic review and meta-analysis of observational studies
Source: Syst Rev. 2024 Feb 23;13:73. doi: 10.1186/s13643-024-02479-3 (PMC10885452; doi:10.1186/s13643-024-02479-3)
Supplement: Supplementary file 1 — Additional file 1. Ovid Medline Search Strategy. [file 13643_2024_2479_MOESM1_ESM.doc]

Database: Ovid MEDLINE(R) ALL <1946 to March 27, 2023>

Search Strategy:

--------------------------------------------------------------------------------

1 exp Frailty/ or Frail*.mp. (39113)

2 Craniotomy.mp. or exp Cranial/ (24524)

3 Cranial Surgery.mp. (653)

4 Neurosurgical.mp. or exp Neurosurgery/ (70074)

5 Post-operative.mp. or exp Postoperative Complications/ (663997)

6 Mortality.mp. (1377148)

7 Length of stay.mp. (146006)

8 Discharge.mp. (246431)

9 Surgical complication.mp. (3175)

10 2 or 3 or 4 (90170)

11 5 or 6 or 7 or 8 or 9 (2194002)

12 1 and 10 and 11 (84)

***************************
