# Supplementary material for: Association between frailty and clinical outcomes in patients undergoing craniotomy—systematic review and meta-analysis of observational studies
Source: Syst Rev. 2024 Feb 23;13:73. doi: 10.1186/s13643-024-02479-3 (PMC10885452; doi:10.1186/s13643-024-02479-3)
Supplement: Supplementary file 2 — Additional file 2. Characteristics of Included Studies. [file 13643_2024_2479_MOESM2_ESM.docx]

*Characteristics of Included Studies*

*Frailty in Geriatric Glioblastoma Patients: A Predictor of Operative Morbidity and Outcome*

Cloney et al 2015 (1)

| *Methods* | This study included all patients age 65 years and older with pathologically confirmed glioblastoma at a single center from 2000 to 2012. Patients with a history of lower grade glioma or with recurrent disease at the time of presentation to the single center were excluded from analysis. Only patients with lobar glioblastoma who underwent  craniotomy were included for calculating complication rates, length of hospitalization, and overall survival. Data were gathered using retrospective chart review. |
| --- | --- |
| *Participants* | This study included all patients aged 65 years and older with pathologically confirmed glioblastoma. Patients with a history of lower grade glioma or with recurrent  disease at the time of presentation were excluded. |
| *Observational parameters* | Frailty was quantified using the mFI 11. This scale accounts for eleven variables, and one point is given for each variable present: difficulty with activities of daily living; history of diabetes mellitus; lung or respiratory disease; congestive heart failure; myocardial infarction; other cardiac disease; arterial hypertension; clouding, delirium, or cognitive impairment; history of transient ischemic attack; history of stroke; and peripheral vascular disease. Comorbid disease burden was measured using the Charlson Comorbidity Index.  Postoperative complications were classified according to the Glioma Outcomes Project system. Neurologic complications were defined as new or worsened neurologic deficits in the immediate postoperative period, including expected or transient deficits. Cardiovascular risk was measured using the Simplified Cardiac Risk Index. |
| Outcomes | In this study outcomes included association between mFI score and the decision to forego surgical resection, the rate of postoperative complications, length of hospital stay,  and overall mortality. |
| Results | Three hundred nineteen patients age 65 years and older were identified, of whom 59 underwent biopsy only and 260 underwent craniotomy for resection. Of the patients undergoing craniotomy, 17 patients with non-lobar glioblastoma were excluded from analysis. Of the 243 patients with lobar glioblastoma who underwent craniotomy, 28 underwent a second resection for recurrent disease, and 3 underwent a third resection, for a total of 274 craniotomies. |

*Frailty as a Predictor of Neurosurgical Outcomes in Brain Tumor Patients*

Harland et al 2020 (2)

| *Methods* | This was a prospective study in patients older than 18. Frailty was preoperatively assessed in 260 patients undergoing surgery for brain tumor resection using a validated scale that assessed weakness, weight loss, exhaustion, low physical activity, and slowed walking speed. Patients were classified as non-frail (score of 0-1), moderately frail (score of 2-3), or frail (score of 4-5). Moderately frail and frail patients were combined for analysis. |
| --- | --- |
| *Participants* | This prospective study enrolled patients  18 years old with a history of brain tumor scheduled for elective resection of tumor at a single center over a three year period. |
| *Observational parameters* | Preoperative assessment of frailty included the 5 components of the Hopkins Frailty Score HFS: shrinking, weakness, exhaustion, low activity, and slowed walking speed. Each domain yielded a dichotomous score of 0 or 1 based on the criteria. Patients were divided into frail (HFS 4e5), moderately frail (HFS 2e3), and non-frail (HFS 0e1) subgroups. The main outcome variables were obtained from the patient’s medical record and included postoperative complications within 30 days of surgery, including mortality; new neurologic deficit; LOS; and discharge to a skilled nursing facility, acute rehabilitation facility, or hospice at 30 days when previously independent. |
| *Outcomes* | This study aimed to determine whether frailty predicts neurosurgical complications in patients with brain tumors and enhances current perioperative risk models. Outcomes included associations of frailty with discharge disposition, length of stay and post-operative complications. |
| *Results* | Preoperative frailty was associated with an increased risk for discharge to a location other than home (10.36; 95% confidence interval, 3.6-30.1), postoperative complications (2.09; 95% confidence interval, 1.09-3.98), and a longer length of stay (1.66; 95% confidence interval, 1.24-2.21) |

*Frailty as a Predictor of Postoperative Complications Following Skull*

*Base Surgery*

*Henry et al 2021 (3)*

| *Methods* | This was a population- level study of the utility of frailty index in patients undergoing skull base surgery. A total of 17,912 patients who underwent skull base procedures were identified using the National Surgical Quality Improvement Program (NSQIP) database from 2005 to 2018. An mFI-5 score was calculated for patients undergoing open skull base surgeries. Multivariate logistic regression analysis was used to evaluate the association of increasing frailty with complications in the 30-day postoperative period. |
| --- | --- |
| *Participants* | The ACS NSQIP participant use data file was queried for all patients undergoing skull base procedures between 2005 and 2018. Over 700 hospitals contributed 30-day morbidity and mortality outcomes on adult patients (≥18 years) undergoing major inpatient and outpatient surgical procedures. Data encompassing a patient’s preoperative risk factors, intraoperative variables, and mortality and morbidity outcomes in the 30-day postoperative period following the index procedure are reported. Patients under the age of 18, those with an ASA  score of six (brain-dead organ donors), trauma cases, and transplant  cases are excluded. Patients undergoing open skull base surgeries were identified  using the principal, concurrent, and other current procedural terminology (CPT). |
| *Observational parameters* | Patient characteristics were collected including age, sex, body mass index (BMI), surgical wound class, operation time, and American Society of Anesthesiologists (ASA) class. Cases with these characteristics and corresponding skull base surgery procedural codes were included in the study. Authors identified and categorized frailty using the 5-factor modified frailty index (mFI-5), validated specifically for use with the ACS NSQIP database. The mFI-5 is calculated using the following variables: non-independent functional status, diabetes mellitus, chronic obstructive pulmonary disease or current pneumonia,  congestive heart failure, and hypertension requiring medication. Non-independent functional status is defined by the NSQIP database as requiring assistance for any activities of daily living, including feeding, dressing, bathing and mobility.  Each factor contributes one point for an mFI-5 score between 0 and 5, with increasing score implying increasing frailty. |
| *Outcomes* | Primary outcome variables included rates of overall complications and life-threatening complications within the 30-day postoperative period. “Overall complications” included any of the following outcomes: superficial surgical site infection (SSI), deep incisional SSI, readmission, mortality, graft/prosthesis/flap failure, deep vein thrombosis (DVT), and life-threatening complications.  Life-threatening postoperative complications were those defined by the Clavien-Dindo classification system. The following Clavien-Dindo grade IV (CDIV) complications are representative of major systemic dysfunction: cerebrovascular accident (CVA), mechanical ventilation for greater than 48 hours, reintubation,  acute renal failure, pulmonary embolism, cardiac arrest, and myocardial infarction. Secondary outcomes included rates of unplanned reoperation and postoperative CSF leak, hydrocephalus, or intracranial hemorrhage requiring surgical intervention. |
| *Results* | Results: A total of 17,912 patients who underwent skull base procedures were identified, with 45.5% of patients having a frailty score of one or greater; 44.9% were male and the mean age was 52.0 ( 16.1 SD) years. Multivariable regression analysis revealed frailty to be an independent predictor of overall complications (odds ratio: 1.325, P < .001), life-threatening complications (OR: 1.428, P < .001), and mortality (OR: 1.453, P < .001). Higher frailty also correlated with increased length of stay. When procedures were stratified by operative location, frailty correlated significantly with overall complications for middle, posterior, and multiple-fossae operations but not the anterior fossa. |

*Predicting Postoperative Outcomes in Brain Tumor Patients With a 5-Factor Modified Frailty Index*

*Huq et al 2021 (4)*

| *Methods* | Authors retrospectively reviewed data for brain tumor patients who underwent primary surgery from 2017 to 2018. Bivariate (ANOVA) and multivariate (logistic and linear regression) analyses assessed the predictive power of the mFI-5 on postoperative outcomes. For bivariate analyses, patients were stratified into 3 categories based on mFI-5 score: zero frailty (mFI-5 = 0), some frailty (mFI-5 = 1), and significant frailty (mFI-5≥2). Clinical and financial outcomes in each of the 3 mFI-5 groups (mFI-5 = 0, mFI-5 = 1, mFI-5 ≥ 2) were presented as mean, standard deviation and compared using one-way analysis of variance (ANOVA), chi squared, or Fisher’s exact tests where appropriate. Multivariate analysis assessing the predictive value of the mFI-5 (assessed linearly with groups of mFI=0, 1, 2, 3, or 4) on outcomes of interest was performed using logistic regression models for binary outcomes (complications, 30-d readmissions) and linear regression models for continuous outcomes (LOS, charges). |
| --- | --- |
| *Participants* | Patient cohort included 1692 adult patients who underwent primary surgery for brain tumors at a single institution between January 1, 2017 and December 31, 2018. |
| *Observational parameters* | Authors extracted data from the institutional database using International Statistical Classification of Diseases and Related Health Problems 10th revision (ICD-10) codes. Demographic and clinical variables collected included age, sex, race, ethnicity, American Society of Anesthesiologists (ASA) classification, and brain tumor diagnosis. Outcome variables included total LOS, ICU LOS, complications (collected using ICD-10 codesas described previously, and 30-d readmissions. Financial outcome variables including pharmacy, imaging, and total charges were provided by the Center for Clinical Data Analysis at the institution. |
| *Outcomes* | Authors examined the predictive power of the mFI-5 on key clinical and financial outcomes (including total length of stay (LOS), intensive care unit (ICU) LOS, complications, charges, and 30-d readmissions) in this patient population. |
| *Results* | Patient cohort had a mean ICU and total LOS of 1.69 and 5.24 d, respectively. Mean complication rates were: pulmonary embolism (PE)/deep vein thrombosis  (DVT) – 7.2%, physiological and metabolic derangement (diabetic ketoacidosis, acute kidney injury) – 1.1%, respiratory failure – 1.6%, sepsis – 1.7%, urinary tract infection (UTI) – 0.5%, and wound infection – 1.4%. Mean pharmacy, imaging, and total charges were $2319, $2304, and $42 331, respectively. Mean 30-d readmission rate was 6.9%. |

*Modified frailty index predicts postoperative outcomes of spontaneous intracerebral hemorrhage*

*Imaoka et al 2018* (5)

| *Methods* | Authors retrospectively reviewed the medical records of 217 consecutive patients who underwent surgery for ICH in two hospitals in Japan from March 2011 to March 2017. Data were extracted from each hospital’s database using the following keywords:  “intracerebral hemorrhage” combined with” craniotomy”, “key hole drainage” or “endoscopic evacuation”. As a rule, the decision to treat ICH surgically in both hospitals followed the stroke guidelines in Japan, that is, a subcortical hemorrhage within 1 cm of the cortical surface, cerebellar hemorrhage of>3 cm in diameter, cerebellar hemorrhage  associated with brainstem compression or hydrocephalus, or the basal ganglia hemorrhage volume of>30 ml associated with a deteriorating level of consciousness. |
| --- | --- |
| *Participants* | 217 consecutive patients who underwent surgery for ICH in two hospitals in Japan.  Only patients with sICH were included in this study. Additional 41 patients were excluded because of loss to follow-up at 6–8 months after sICH, leaving 156 patients meeting the inclusion criteria. |
| *Observational parameters* | The CSHA-FI has been mapped to 11 variables contained in the American College of Surgeons National Surgical Quality Improvement Program (NSQIP) database to develop a  modified frailty index (mFI). Data collected included sex; mFI variables; antiplatelet agents used; anticoagulant agents used; hematoma location (subcortex, basal ganglia  or thalamus, or cerebellum); intraventricular hemorrhage (IVH) and operative method (craniotomy or minimally invasive surgery). |
| *Outcomes* | Patients and methods: Outcome measures included an unfavorable outcome (modified Rankin Scale score of 4–6) or mortality at 6–8 months after hemorrhage. The prognostic ability of mFI was assessed by comparing adjusted and nonadjusted effects with the Hemphill’s ICH score. The performance of the ICH score combined with mFI  was assessed for discriminative ability. |
| *Results* | Results: In total, 156 patients satisfied the inclusion criteria. Multivariate analyses revealed that higher mFI was significantly associated with an unfavorable outcome (p-value=0.004) and mortality (p-value<0.001). |

*The role of frailty in geriatric cranial neurosurgery for primary central nervous system neoplasms*

*Shahrestani et al 2020 (6)*

| *Methods* | The authors conducted a retrospective cohort study of geriatric patients receiving cranial neurosurgery for a primary CNS neoplasm between 2010 and 2017 by using the Nationwide Readmission Database. Demographics and frailty were queried at primary admission, and readmissions were analyzed at 30-, 90-, and 180-day intervals. Complications  of interest included infection, anemia, infarction, kidney injury, CSF leak, urinary tract infection, and mortality. Nearest-neighbor propensity score matching for demographics was implemented to identify nonfrail control patients with similar diagnoses and procedures. The analysis used Welch two-sample t-tests for continuous variables and chi-square test with odds ratios. |
| --- | --- |
| *Participants* | Participants were patients older than 65 patients who were receiving cranial neurosurgery for a primary CNS neoplasm between 2010 and 2017 by using the Nationwide Readmission Database. Charlson Comorbidity Index (CCI) scores were collected for each patient and used to develop 10-year survival estimates for all patients. |
| *Observational parameters* | In this study authors used the Healthcare Cost and Utilization Project (HCUP) NRD from the years 2010 to 2017. The NRD is a large yearly database that publishes national  information regarding inpatient demographics, diagnoses, procedures, and readmissions. Charlson Comorbidity Index (CCI) scores were collected for each patient and used to develop 10-year survival estimates for all patients. |
| *Outcomes* | Demographics and frailty were queried at primary admission, and readmissions were analyzed at 30-, 90-, and 180-day intervals. Complications of interest included infection, anemia, infarction, kidney injury, CSF leak, urinary tract infection, and mortality.  Nearest-neighbor propensity score matching for demographics was implemented to identify nonfrail control patients with similar diagnoses and procedures. The analysis used Welch two-sample t-tests for continuous variables and chi-square test with odds ratios. |
| *Results* | A total of 6713 frail patients and 6629 nonfrail patients were identified at primary admission. At primary admission, frail geriatric patients undergoing cranial neurosurgery had increased odds of developing acute posthemorrhagic anemia (OR 1.56, 95% CI 1.23–1.98; p = 0.00020); acute infection (OR 3.16, 95% CI 1.70–6.36; p = 0.00022);  acute kidney injury (OR 1.32, 95% CI 1.07–1.62; p = 0.0088); urinary tract infection prior to discharge (OR 1.97, 95% CI 1.71–2.29; p < 0.0001); acute postoperative cerebral infarction (OR 1.57, 95% CI 1.17–2.11; p = 0.0026); and mortality (OR 1.64, 95% CI 1.22–2.24; p = 0.0012) compared to nonfrail geriatric patients receiving the same procedure. In  addition, frail patients had a significantly increased inpatient length of stay (p < 0.0001) and all-payer hospital cost (p <0.0001) compared to nonfrail patients at the time of primary admission. However, no significant difference was found between frail and nonfrail patients with regard to rates of infection, thromboembolism, CSF leak, dural tear, cerebral infarction, acute kidney injury, and mortality at all readmission time points. |

*Frailty predicts worse outcomes after intracranial meningioma surgery irrespective of existing prognostic*

*factors*

*Theriault 2020* (7)

| *Methods* | This is a single-center retrospective cohort study of patients who underwent intracranial meningioma resection between August 2012 and May 2018. Seventy-six patients met the inclusion criteria. |
| --- | --- |
| *Participants* | Seventy-six patients, who had undergone craniotomy for resection of an intracranial meningioma between August 2012 and May 2018, met the study’s inclusion criteria. The cohort was 72.6% female, with a mean age of 55.8  ± 1.8 years and a mean BMI of 27.9 ± 0.64. |
| *Observational parameters* | The modified Frailty Index (mFI) was used to measure frailty in this study. Because the required components of this index are routinely included in patient charts, the  mFI is conducive to a retrospective chart review, whereas other frailty measurements requiring specific assessments were not available for this study. The patients’ mFIs were determined as previously described by reviewing patient  charts and tallying how many mFI variables were identified in each patient. Each mFI variable is equally weighted and counted as 1. The patient cohort had an overall low mFI (0.86), and thus patients were categorized as either non-frail (mFI = 0) or frail (mFI ≥ 1). |
| *Outcomes* | The primary outcomes were hospital LOS, discharge location, readmission  rates, and reoperation rates. Authors examined frailty’s effect on these primary outcomes. Secondary analyses examining the effect of patient age (≥ 65 vs < 65 years), sex (male vs female), BMI (≥ 30 vs < 30), tumor size (> 3.5 vs  < 3.5 cm), or tumor location (skull base vs non–skull base) were conducted on the same outcomes. |
| Results | Frailty was associated with increased hospital LOS (p = 0.0218), increased reoperation rate (p = 0.029), and discharge to a higher level of care: an inpatient rehabilitation facility or a skilled nursing facility (p = 0.0002). After multivariable  analysis, frailty was determined to be an independent risk factor for increased LOS, worse discharge disposition, and subsequent readmission. |

*The modified frailty index and 30-day adverse events in oncologic Neurosurgery*

*Youngerman et al 2017 (8)*

| *Methods* | Authors identified patients in the National Surgical Quality Improvement  Program who underwent oncologic neurosurgery procedures between 2008 and 2012. The mFI 11, ranging from 0 to 1, was calculated as the proportion of 11 possible risk factors present. They assessed the associations between mFI and 30-day mortality, neurologic and medical complications, prolonged length of stay, and unfavorable discharge in univariate and multivariable analyses and compare the index to established risk stratification techniques. A total of 9149 patients were identified. |
| --- | --- |
| *Participants* | Authors identified patients who underwent neurosurgical procedures for intracranial neoplasms between 2008 and 2012. Patients were included if they had a Current Procedural Terminology (CPT) procedure code representing a surgery classified as supratentorial brain, supratentorial dural, or infratentorial. |
| *Observational parameters* | The mFI was calculated using 11 variables from the Canadian Study of Health and Aging (CSHA) Frailty Index that were matched to variables in NSQIP. Each variable was scored one point in the numerator for every condition present. The mFI in this study ranged from 0 to 1 with higher scores representing increasing frailty. |
| *Outcomes* | The outcomes measured were 30-day mortality, 30-day severe medical complications, 30-day severe neurologic complications, 30-day any complication, extended length of stay (LOS), and unfavorable disposition. Mortality was defined as death within 30 days of the index procedure. Severe medical complications were defined according to the Clavian class IV categorization. Any complication included the occurrence of a severe medical  or neurologic complication, or a wound complication, pneumonia, acute renal failure,  urinary tract infection, deep vein thrombosis, or sepsis. Prolonged length of stay was defined as hospitalizations lasting longer than 7 days. Unfavorable disposition was  defined as a discharge destination other than home or a facility with a higher level of care than before admission. |
| *Results* | Fewer surgeries were performed at increasing levels of frailty. The majority of patients  had no frailty (51.5%), while 21.3% had low, 21.5% intermediate, and 5.7% high frailty. Transsphenoidal surgery patients were more likely to have low preoperative frailty  (35.1%) than the full cohort (21.3%). Patients with benign primary brain tumors and primary cranial nerve tumors were also less frail. Compared to the full cohort, patients  were more likely to have intermediate or high frailty if they had secondary malignant brain tumors, older age, were Black compared to White, or had higher preoperative  ASA class, obesity, bleeding disorders, dependent functional status, hemiplegia, ventilator dependence, sepsis, low albumin (< 3.5), weight loss, blood transfusion,  corticosteroid use, chemotherapy in the past month, or radiotherapy in the past 90 days.  In the univariate analysis increased mFI was associated with stepwise increases in the rates of mortality, severe medical complications, prolonged length of stay, and unfavorable discharge. |

*Frailty and outcomes after craniotomy for brain tumor*

Sastry et al 2020 (9)

| *Methods* | A retrospective cohort study was conducted on 20,333 adult patients undergoing elective craniotomy for tumor resection in the 2012–2018 ACS-NSQIP Participant  Use File. Multivariate logistic regression was performed using all covariates deemed eligible through clinical and statistical significance. 6,249 patients (30.7%) were low-frailty and 2,148 patients (10.6%) were medium-to-high frailty. In multivariate logistic regression adjusting for age, gender, BMI, ASA classification, smoking status, dyspnea, significant pre-operative weight loss, chronic steroid use, bleeding disorder,  tumor type, and operative time, low frailty was associated with increased adjusted odds ratio of major complication (1.41, 95% CI: 1.23–1.60, p < 0.001), discharge destination other than home (1.32, 95% CI: 1.20–1.46, p < 0.001), 30-day readmission (1.29, 95% CI: 1.15–1.44, p < 0.001), and 30-day mortality (1.87, 95% CI: 1.41–2.47, p < 0.001). Moderate-to-high frailty was also associated with increased adjusted odds of major complication (1.61, 95% CI: 1.35–1.92, p < 0.001), discharge destination other than  home (1.80, 95% CI: 1.58–2.05), 30-day readmission (1.39, 95% CI: 1.19–1.62, p < 0.001), and 30-day mortality (2.42, 95% CI: 1.74–3.38, p < 0.001). |
| --- | --- |
| *Participants* | Authors analyzed adult (age greater than 18 years) patients undergoing elective cranial surgery for tumor. Authors identified patients through a combination of Current Procedural Terminology (CPT) and postoperative International Classification of Disease (ICD) codes. Inclusion and exclusion criteria were designed to select for the most medically-optimized patient population we could reasonably achieve using NSQIP. |
| *Observational parameters* | mFI-5 and Outcomes: The mFI-5 score was calculated by adding the number of present variables for a given patient and dividing by 5. Scores were then classified as non-frailty (mFI-5 = 0), low frailty (mFI = 0.2), or medium-to-high frailty (mFI > 0.2), in concordance  with other studies that have used mFI-5. |
| *Outcomes* | Authors assessed the relationship between frailty and the incidence of major post-operative complication, discharge destination other than home, 30-day readmission, and 30-day mortality after elective craniotomy for brain tumor resection. |
| *Results* | In multivariate logistic regression adjusting for age, gender, BMI, ASA classification,  smoking status, dyspnea, significant pre-operative weight loss, chronic steroid use, bleeding disorder,tumor type, and operative time, low frailty was associated with increased adjusted odds ratio of major complication (1.41, 95% CI: 1.23–1.60, p < 0.001), discharge destination other than home (1.32, 95% CI: 1.20–1.46, p < 0.001), 30-day readmission (1.29, 95% CI: 1.15–1.44, p < 0.001), and 30-day mortality  (1.87, 95% CI: 1.41–2.47, p < 0.001). Moderate-to-high frailty was also associated with increased adjusted odds of major complication (1.61, 95% CI: 1.35–1.92, p < 0.001), discharge destination other than home (1.80, 95% CI: 1.58–2.05), 30-day readmission (1.39, 95% CI: 1.19–1.62, p < 0.001), and 30-day mortality (2.42, 95% CI: 1.74–3.38, p < 0.001); |

References:

1. Cloney M, D'Amico R, Lebovic J, Nazarian M, Zacharia BE, Sisti MB, et al. Frailty in Geriatric Glioblastoma Patients: A Predictor of Operative Morbidity and Outcome. World Neurosurg. 2016;89:362-7.

2. Harland TA, Wang M, Gunaydin D, Fringuello A, Freeman J, Hosokawa PW, et al. Frailty as a Predictor of Neurosurgical Outcomes in Brain Tumor Patients. World Neurosurg. 2020;133:e813-e8.

3. Henry RK, Reeves RA, Wackym PA, Ahmed OH, Hanft SJ, Kwong KM. Frailty as a Predictor of Postoperative Complications Following Skull Base Surgery. Laryngoscope. 2021;131(9):1977-84.

4. Huq S, Khalafallah AM, Jimenez AE, Gami A, Lam S, Ruiz-Cardozo MA, et al. Predicting Postoperative Outcomes in Brain Tumor Patients With a 5-Factor Modified Frailty Index. Neurosurgery. 2020;88(1):147-54.

5. Imaoka Y, Kawano T, Hashiguchi A, Fujimoto K, Yamamoto K, Nishi T, et al. Modified frailty index predicts postoperative outcomes of spontaneous intracerebral hemorrhage. Clinical Neurology and Neurosurgery. 2018;175.

6. Shahrestani S, Lehrich BM, Tafreshi AR, Brown NJ, Lien BV, Ransom S, et al. The role of frailty in geriatric cranial neurosurgery for primary central nervous system neoplasms. Neurosurg Focus. 2020;49(4):E15.

7. Theriault BC, Pazniokas J, Adkoli AS, Cho EK, Rao N, Schmidt M, et al. Frailty predicts worse outcomes after intracranial meningioma surgery irrespective of existing prognostic factors. Neurosurg Focus. 2020;49(4):E16.

8. Youngerman BE, Neugut AI, Yang J, Hershman DL, Wright JD, Bruce JN. The modified frailty index and 30-day adverse events in oncologic neurosurgery. J Neurooncol. 2018;136(1):197-206.

9. Sastry RA, Pertsch NJ, Tang O, Shao B, Toms SA, Weil RJ. Frailty and outcomes after craniotomy for brain tumor. J Clin Neurosci. 2020;81:95-100.
