# Supplementary material for: Association between frailty and clinical outcomes in patients undergoing craniotomy—systematic review and meta-analysis of observational studies
Source: Syst Rev. 2024 Feb 23;13:73. doi: 10.1186/s13643-024-02479-3 (PMC10885452; doi:10.1186/s13643-024-02479-3)
Supplement: Supplementary file 4 — Additional file 4. Excluded studies with reasons. [file 13643_2024_2479_MOESM4_ESM.docx]

Excluded studies with reasons

1. Adams, P, Ghanem, T, Stachler, R, Hall, F, Velanovich, V, and Rubinfeld, I. Frailty as a predictor of morbidity and mortality in inpatient head and neck surgery. *JAMA Otolaryngol Head Neck Surg*. (2013) 139:783–9. doi: 10.1001/jamaoto.2013.3969

Outisde the scope of study.

1. Asemota, AO, and Gallia, L. Impact of frailty on short-term outcomes in patients undergoing transsphenoidal pituitary surgery. *J Neurosurg*. (2019) 132:360–70. doi: 10.3171/2018.8.JNS181875

Outside the scope of study.

1. Bonney, PA, Chartrain, AG, Briggs, RG, Jarvis, CA, Ding, L, Mack, WJ, et al. Frailty is associated with in-hospital morbidity and nonroutine disposition in brain tumor patients undergoing craniotomy. *World Neurosurg*. (2021) 146:e1045–53. doi: 10.1016/j.wneu.2020.11.083

This study was unclear whether it studied primary admissions only or repeat surgery. There was overlap with Shahrestani where primary admissions only were included.

1. Assessment of Frailty in Predicting Surgical Outcomes in Patients with Chronic Subdural

Hematomas: Retrospective Chart Review

Mohamad Kesserwan, Bradley Bergin, Arunachala Trivedi, Husain Shakil, Amanda Martyniuk, Radwan Takroni, Ekkehard Kasper, Paul Engels, Forough Farrokhyar, Sunjay Sharma

1. Importance of frailty evaluation in the prediction of the prognosis of patients with chronic subdural hematoma.

Kiyoharu Shimizu,1 Takashi Sadatomo,1 Takeshi Hara,1 Shumpei Onishi,1 Kiyoshi Yuki1 and

Kaoru Kurisu2

Outside the scope of study.

1. Worse cranial neurosurgical outcomes predicted by increasing frailty in patients with interhospital transfer status: Analysis of 47,736 patients from the National Surgical Quality Improvement Program (NSQIP) 2015–2019 Alexander J. Kassicieh , Samantha Varela , Kavelin Rumalla , Syed Faraz Kazim , Kyril L. Cole , Desna V. Ghatalia , Meic H. Schmidt , Christian A. Bowers.

Outside the scope of study.

1. Khalafallah, AM, Huq, S, Jimenez, AE, Brem, H, and Mukherjee, D. The 5-factor modified frailty index: an effective predictor of mortality in brain tumor patients. *J Neurosurg*. (2020) 135:1–9. doi: 10.3171/2020.5.JNS20766

Comparison of utility, rather than an original study.

8 . Shahrestani, S, Ballatori, AM, Chen, XT, Ton, A, Strickland, BA, Brunswick, A, et al. Analysis of modifiable and nonmodifiable risk factors in patients undergoing pituitary surgery. *J Neurosurg*. (2020) 134:1–8. doi: 10.3171/2020.4.JNS20417

Pituitary Surgery

9. Pitts, KD, Arteaga, AA, Stevens, BP, White, WC, Su, D, Spankovich, C, et al. Frailty as a predictor of postoperative outcomes among patients with head and neck cancer. *Otolaryngol Head Neck Surg*. (2019) 160:664–71. doi: 10.1177/0194599818825466

Head and Neck Surgery

10. Segal, JB, Chang, H-Y, Du, Y, Walston, JD, Carlson, MC, and Varadhan, R. Development of a claims-based frailty indicator anchored to a well-established frailty phenotype. *Med Care*. (2017) 55:716–22. doi: 10.1097/MLR.0000000000000729

Development of new indicator

11. Huq S, Liu J, Romano R, Seal S, Khalafallah AM, Walston JD, et al.

Frailty in Patients Undergoing Surgery for Brain Tumors: A Systematic Review of the Literature. World Neurosurg. 2022;166:268-78.e8.

Narrative Systematic review

12. A comparative analysis of the Hospital Frailty Risk Score in predicting postoperative outcomes among intracranial tumor patients.

Jimenez AE, Liu J, Cicalese KV, Jimenez MA, Porras JL, Azad TD, Jackson C, Gallia GL, Bettegowda C, Weingart J, Mukherjee D

Journal of Neurosurgery. 1-10, 2022 Dec 16.

Outside the scope of study.

13. Frailty is a risk factor for intracranial abscess and is associated with longer length of stay: a retrospective single institution case-control study.

Dominguez JF, Sursal T, Kazim SF, Ng C, Vazquez S, DAS A, Naftchi A, Spirollari E, Elkun Y, Gatzoflias S, Ampie L, Feldstein E, Uddin A, Damodara N, Hanft SJ, Gandhi CD, Bowers CA

Journal of Neurosurgical Sciences. 2022 Apr 13.

14. Social determinants of health and the prediction of 90-day mortality among brain tumor patients.

Jimenez AE, Cicalese KV, Chakravarti S, Porras JL, Azad TD, Jackson CM, Gallia GL, Bettegowda C, Weingart J, Mukherjee D

Journal of Neurosurgery. 1-9, 2022 Mar 11.

15. Frailty as a predictor of neurosurgical outcomes in brain tumor patients: A systematic review and meta-analysis.

Zhu J, Qiu X, Ji C, Wang F, Tao A, Chen L

Frontiers in psychiatry Frontiers Research Foundation. 14:1126123, 2023.

Zhu, Jinfeng, Qiu, Xichenhui, Ji, Cuiling, Wang, Fang, Tao, An, Chen, Lu

16. Surgical and Peri-Operative Considerations for Brain Metastases.

Gupta S, Dawood H, Giantini Larsen A, Fandino L, Knelson EH, Smith TR, Lee EQ, Aizer A, Dunn IF, Bi WL

Frontiers in Oncology. 11:662943, 2021.

Gupta, Saksham, Dawood, Hassan, Giantini Larsen, Alexandra, Fandino, Luis, Knelson, Erik H, Smith, Timothy R, Lee, Eudocia Q, Aizer, Ayal, Dunn, Ian F, Bi, Wenya Linda

17. Impact of Comorbidities and Frailty on Early Shunt Failure in Geriatric Patients With Normal Pressure Hydrocephalus.

Hadjiathanasiou A, Kilinc F, Behmanesh B, Bernstock J, Guresir E, Heimann M, Konczalla J, Scharnbock E, Schneider M, Weinhold L, Seifert V, Vatter H, Gessler F, Schuss P

Frontiers in Medicine. 7:596270, 2020.

18. Preoperative risk model for perioperative stroke after intracranial tumor resection: ACS NSQIP analysis of 30,951 cases.

Kassicieh AJ, Rumalla K, Kazim SF, Asserson DB, Schmidt MH, Bowers CA

Neurosurgical Focus. 53(6):E9, 2022 12.

19. Utility of hospital frailty risk score for predicting postoperative outcomes in craniopharyngioma.

Peterson R, Kandregula S, Jee E, Guthikonda B

Journal of Neuro-Oncology. 159(1):185-193, 2022 Aug.

[Journal Article]

UI: 35723816

Title Comment

Comment in: J Neurooncol. 2022 Oct;160(1):273-275

20. The Impact of Frailty on Traumatic Brain Injury Outcomes: An Analysis of 691 821 Nationwide Cases.

Tang OY, Shao B, Kimata AR, Sastry RA, Wu J, Asaad WF

Neurosurgery. 91(5):808-820, 2022 11 01.

21. Preoperative frailty measured by risk analysis index predicts complications and poor discharge outcomes after Brain Tumor Resection in a large multi-center analysis.

Thommen R, Kazim SF, Rumalla K, Kassicieh AJ, Kalakoti P, Schmidt MH, McKee RG, Hall DE, Miskimins RJ, Bowers CA

Journal of Neuro-Oncology. 160(2):285-297, 2022 Nov.

22.Factors Predicting Frailty Among Postoperative Brain Tumor Patients.

Mungngam C, Utriyaprasit K, Tankumpuan T, Sitthinamsuwan B

Journal of Neuroscience Nursing. 54(6):240-244, 2022 Dec 01.

23. Patient frailty association with cerebral arteriovenous malformation microsurgical outcomes and development of custom risk stratification score: an analysis of 16,721 nationwide admissions.

Tang OY, Bajaj AI, Zhao K, Liu JK

Neurosurgical Focus. 53(1):E14, 2022 07.

24. Worse cranial neurosurgical outcomes predicted by increasing frailty in patients with interhospital transfer status: Analysis of 47,736 patients from the National Surgical Quality Improvement Program (NSQIP) 2015-2019.

Kassicieh AJ, Varela S, Rumalla K, Kazim SF, Cole KL, Ghatalia DV, Schmidt MH, Bowers CA

Clinical Neurology & Neurosurgery. 221:107383, 2022 10.

25. Use of the 5-Factor Modified Frailty Index to Predict Hospital-Acquired Infections and Length of Stay Among Neurotrauma Patients Undergoing Emergent Craniotomy/Craniectomy.

Cole KL, Kurudza E, Rahman M, Kazim SF, Schmidt MH, Bowers CA, Menacho ST

World Neurosurgery. 164:e1143-e1152, 2022 08.

Limited scope of study.

22.Relevance of presenting risks of frailty, sarcopaenia and osteopaenia to outcomes from aneurysmal subarachnoid haemorrhage.

Lim JX, Lim YG, Kumar A, Cheong TM, Han JX, Chen MW, Wen D, Lim W, Ng IHB, Ng VYP, Kirollos RW, Keong NCH

BMC Geriatrics. 22(1):333, 2022 04 16.

Ineligible according to study criteria.

24. Association of Patient Frailty With Vestibular Schwannoma Resection Outcomes and Machine Learning Development of a Vestibular Schwannoma Risk Stratification Score.

Tang OY, Bajaj AI, Zhao K, Rivera Perla KM, Ying YM, Jyung RW, Liu JK

Neurosurgery. 91(2):312-321, 2022 08 01.

Limited scope of study.

25. Association of baseline frailty status with clinical outcome following aneurysmal subarachnoid hemorrhage.

Dicpinigaitis AJ, McIntyre MK, Al-Mufti F, Kazim SF, Li B, Schmidt MH, Gandhi CD, Cole CD, Bowers CA

Journal of Stroke & Cerebrovascular Diseases. 31(5):106394, 2022 May.

Ineligible according to study criteria.

26. Worse Pituitary Adenoma Surgical Outcomes Predicted by Increasing Frailty, Not Age.

Thommen R, Kazim SF, Cole KL, Olson GT, Shama L, Lovato CM, Gonzales KM, Dicpinigaitis AJ, Couldwell WT, Mckee RG, Cole CD, Schmidt MH, Bowers CA

World Neurosurgery. 161:e347-e354, 2022 05.

Limited scope of study.

27.Comparative associations of baseline frailty status and age with postoperative mortality and duration of hospital stay following metastatic brain tumor resection.

Dicpinigaitis AJ, Hanft S, Cooper JB, Gandhi CD, Kazim SF, Schmidt MH, Al-Mufti F, Bowers CA

Limited scope of study (metastasis only).

28. Does general comorbidity impact the postoperative outcomes after surgery for large and giant petroclival meningiomas?.

Roux A, Troude L, Baucher G, Bernard F, Pallud J, Roche PH

Neurosurgical Review. 45(1):617-626, 2022 Feb.

Limited scope of study.

29. Patient-Specific Factors Drive Intensive Care Unit and Total Hospital Length of Stay in Operative Patients with Brain Tumor.

Jimenez AE, Shah PP, Khalafallah AM, Huq S, Porras JL, Jackson CM, Gallia G, Bettegowda C, Weingart J, Suarez JI, Brem H, Mukherjee D

World Neurosurgery. 153:e338-e348, 2021 09.

Limited scope of study.

29. Association of preoperative frailty with postoperative delirium after elective brain tumor resection: Retrospective analysis of a prospective cohort.

Wang CM, Ma YL, Yang XY, Ji RQ, Gu WJ, Zhou JX

Surgery. 170(6):1763-1769, 2021 12.

Limited scope of study.

30. Frailty Is Associated with In-Hospital Morbidity and Nonroutine Disposition in Brain Tumor Patients Undergoing Craniotomy.

Bonney PA, Chartrain AG, Briggs RG, Jarvis CA, Ding L, Mack WJ, Zada G, Attenello FA

World Neurosurgery. 146:e1045-e1053, 2021 02.

31.Frailty and Outcomes after Craniotomy or Craniectomy for Atraumatic Chronic Subdural Hematoma.

Sastry RA, Pertsch N, Tang O, Shao B, Toms SA, Weil RJ

World Neurosurgery. 145:e242-e251, 2021 01.

Does not meet entry criteria.

32.The immense heterogeneity of frailty in neurosurgery: a systematic literature review.

Pazniokas J, Gandhi C, Theriault B, Schmidt M, Cole C, Al-Mufti F, Santarelli J, Bowers CA

Neurosurgical Review. 44(1):189-201, 2021 Feb.

Outside the scope of study.

33. The Effect of Frailty and Patient Comorbidities on Outcomes After Acute Subdural Hemorrhage: A Preliminary Analysis.

Rawanduzy C, McIntyre MK, Afridi A, Honig J, Halabi M, Hehir J, Schmidt M, Cole C, Miller I, Gandhi C, Al-Mufti F, Bowers C

World Neurosurgery. 143:e285-e293, 2020 11.

This study does not meet entry criteria.

34. Risk factors associated with early adverse outcomes following craniotomy for malignant glioma in older adults.

Rahmani R, Tomlinson SB, Santangelo G, Warren KT, Schmidt T, Walter KA, Vates GE

Journal of Geriatric Oncology. 11(4):694-700, 2020 05.

Limited scope of study.

35. Thirty-Day Mortality and Survival in Elderly Patients Undergoing Neurosurgery.

Bligh ER, Sinha P, Smith D, Al-Tamimi YZ

World Neurosurgery. 133:e646-e652, 2020 Jan.

Limited scope of study.

36. Factors affecting patient flow in a neurosurgery department.

Irvine S, Awan M, Chharawala F, Bhagawati D, Lawrance N, Peck G, Peterson D, Banerjee S, Camp S

Annals of the Royal College of Surgeons of England. 102(1):18-24, 2020 Jan.

Limited scope of study.

37. Predictors of Successful Discharge of Patients on Postoperative Day 1 After Craniotomy for Brain Tumor.

Richardson AM, McCarthy DJ, Sandhu J, Mayrand R, Guerrero C, Rosenberg C, Gernsback JE, Komotar R, Ivan M

World Neurosurgery. 126:e869-e877, 2019 Jun.

Limited scope of study.

38. Frailty Cost: Economic Impact of Frailty in the Elective Surgical Patient.

Wilkes JG, Evans JL, Prato BS, Hess SA, MacGillivray DC, Fitzgerald TL

Journal of the American College of Surgeons. 228(6):861-870, 2019 06.

Out of scope of study.

39. Factors Related to Frailty Associated with Clinical Deterioration After Meningioma Surgery in the Elderly.

Isobe N, Ikawa F, Tominaga A, Kuroki K, Sadatomo T, Mizoue T, Hamasaki O, Matsushige T, Abiko M, Mitsuhara T, Kinoshita Y, Takeda M, Kurisu K

World Neurosurgery. 119:e167-e173, 2018 Nov.

Limited scope of study.

40. Preoperative Frailty Score for 30-Day Morbidity and Mortality After Cranial Neurosurgery.

Tomlinson SB, Piper K, Kimmell KT, Vates GE

World Neurosurgery. 107:959-965, 2017 Nov.

Limited scope of study.
