# Supplementary material for: Association between frailty and clinical outcomes in patients undergoing craniotomy—systematic review and meta-analysis of observational studies
Source: Syst Rev. 2024 Feb 23;13:73. doi: 10.1186/s13643-024-02479-3 (PMC10885452; doi:10.1186/s13643-024-02479-3)
Supplement: Supplementary file 5 — Additional file 5. Supplementary Data. [file 13643_2024_2479_MOESM5_ESM.docx]

***Supplementary Data***

***Funnel Plot Assessment of Publication Bias***

***
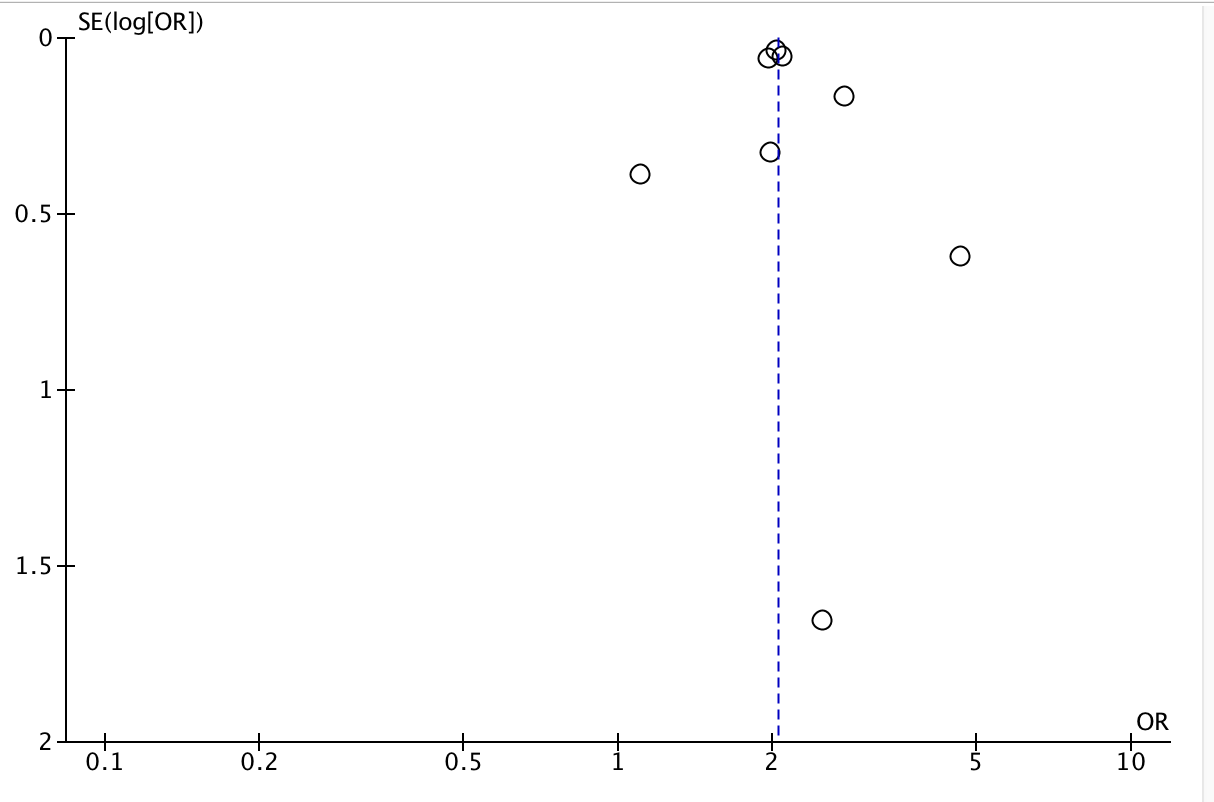
***

***Secondary outcomes***

*Length of hospital stay in frail patients compared to non-frail cohort*

***
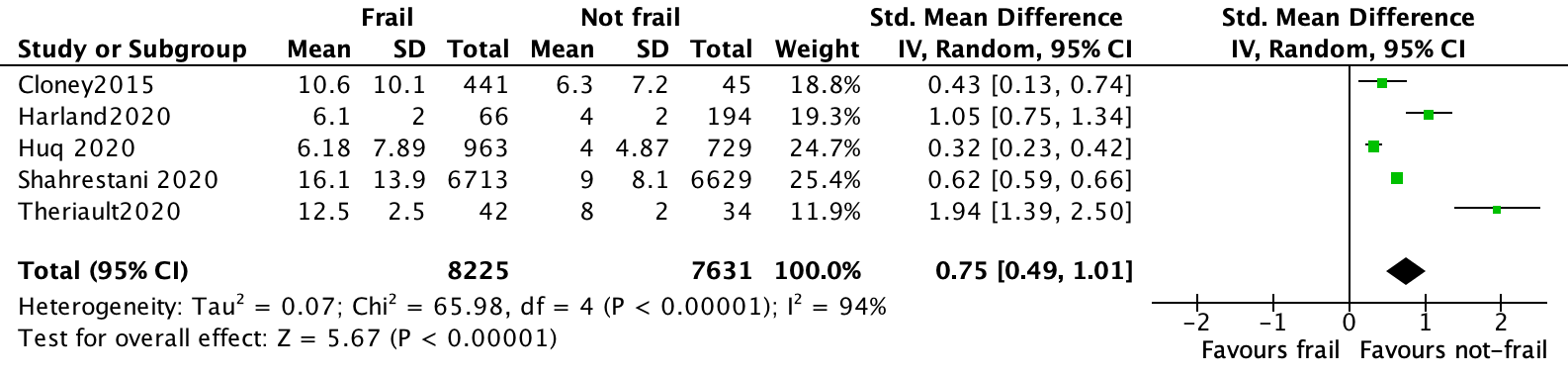
***

*Discharge to a location other than home*


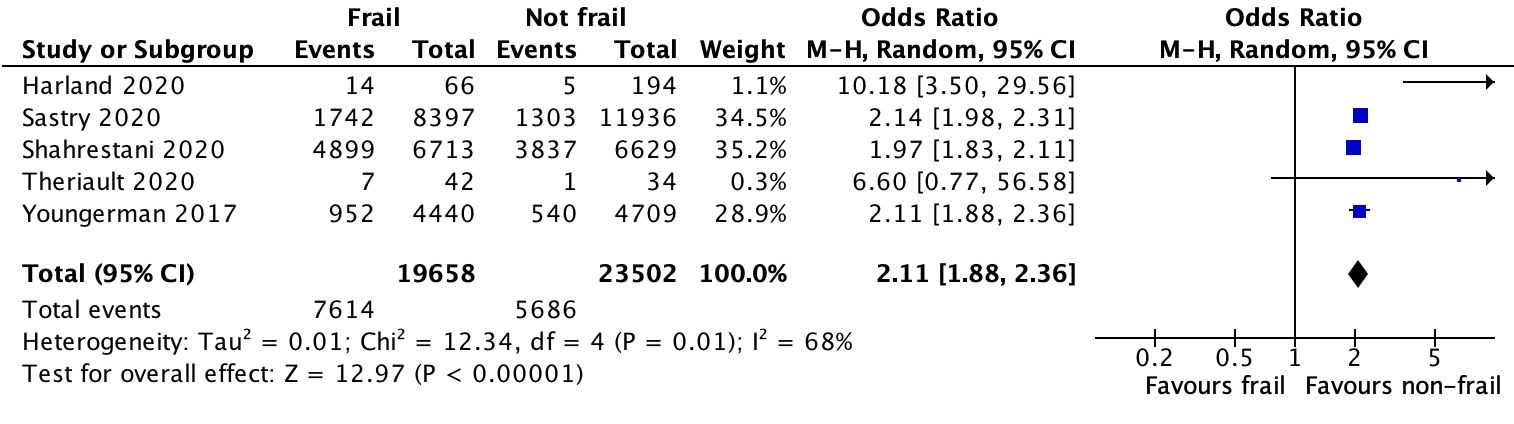


*Hospital readmission in frail compared to non-frail patients*

***
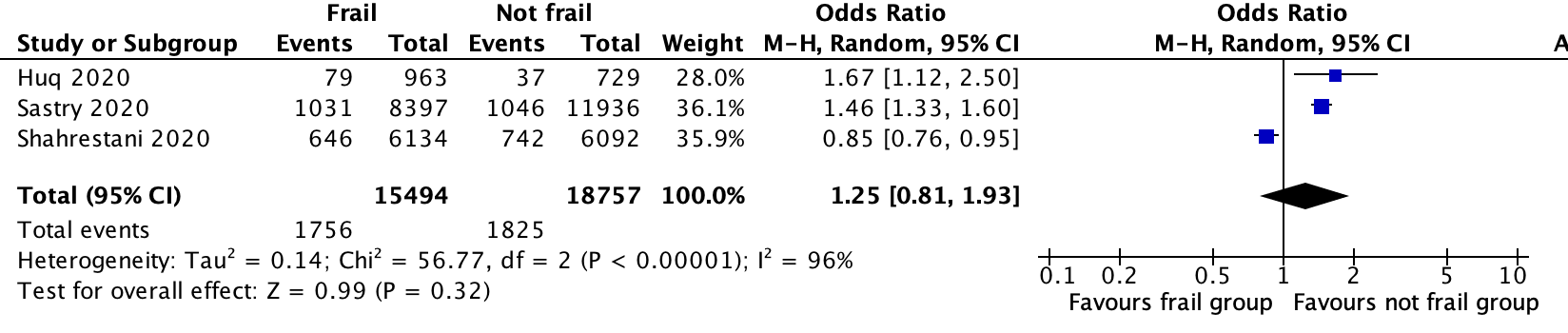
***

***Sensitivity analysis***

*Retrospective studies only*

**
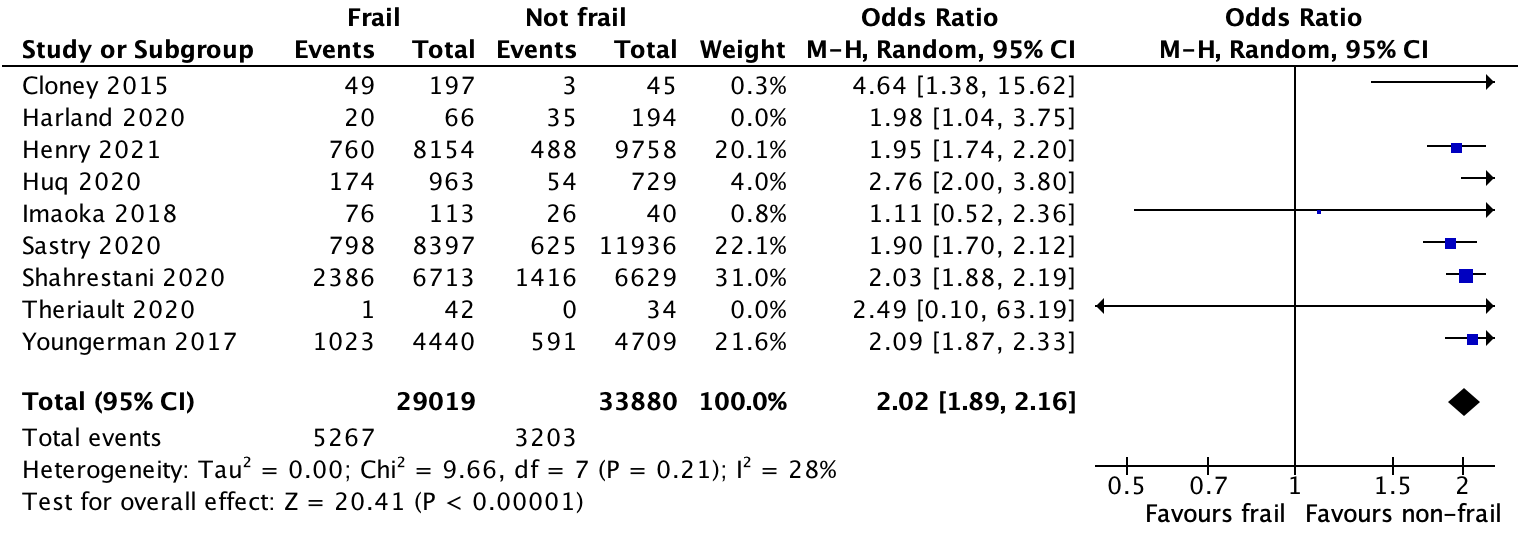
**

*Analysis excluding studies at high risk of bias*

***
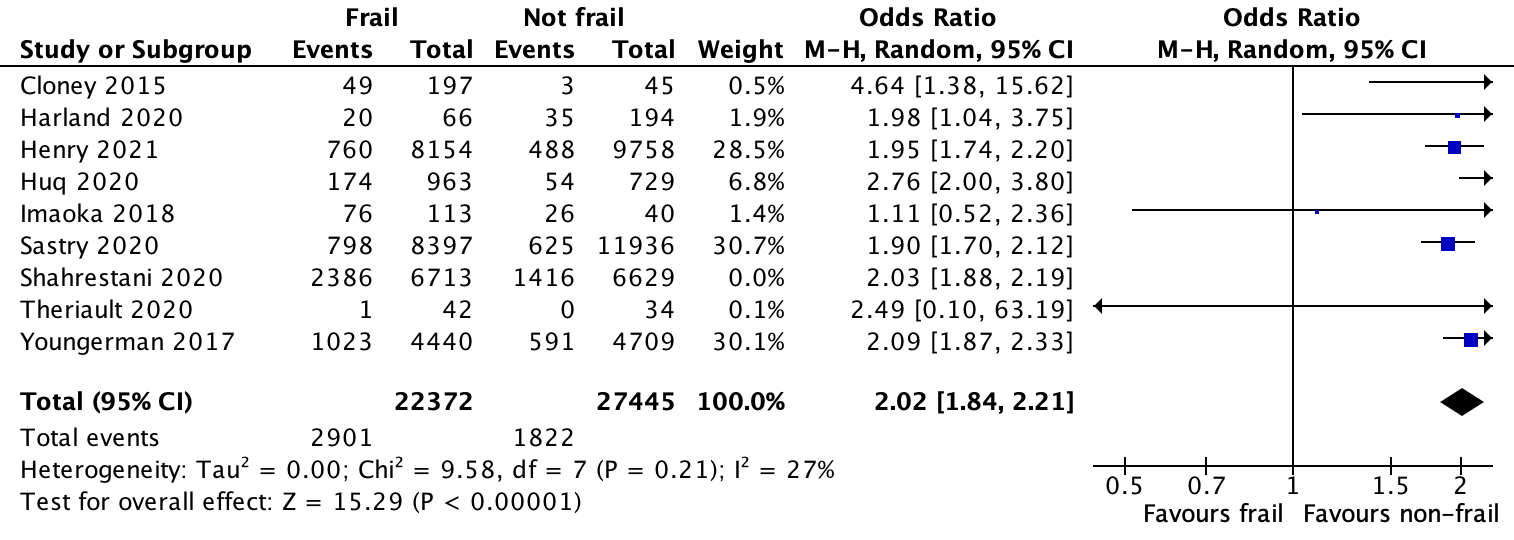
***

***Subgroup analysis***

*Clavien-Dindo complications in patients undergoing tumour surgery*


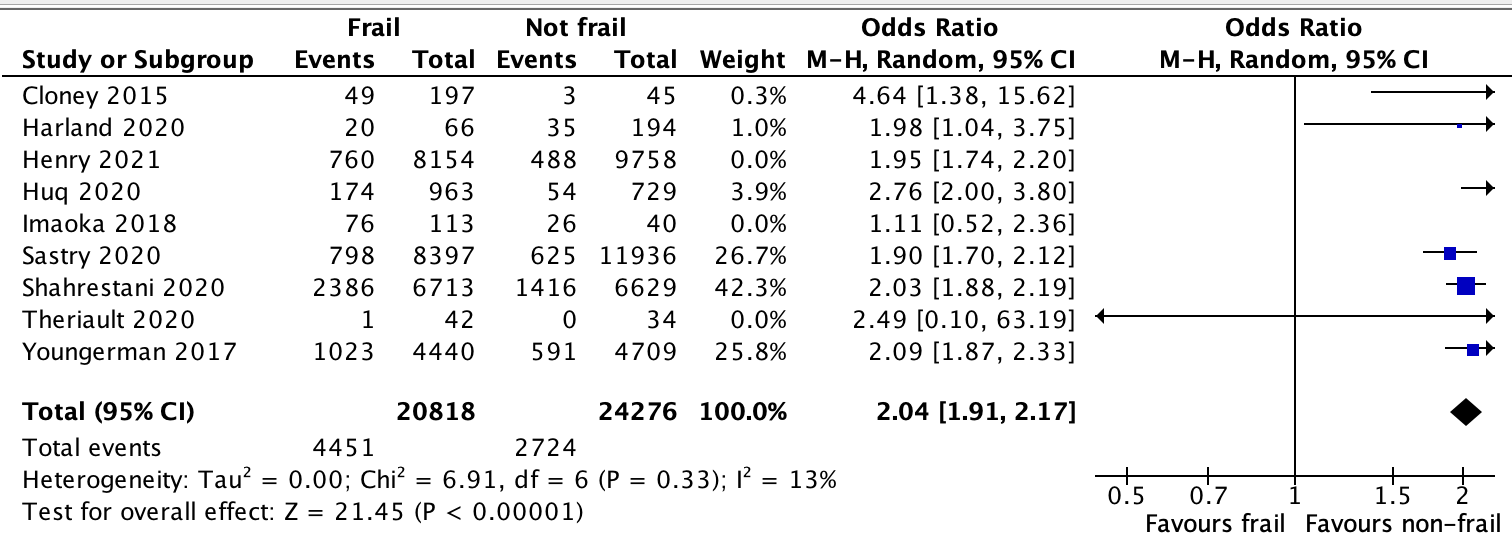


*Clavien-Dindo complications in patients undergoing non-tumour surgery*

***
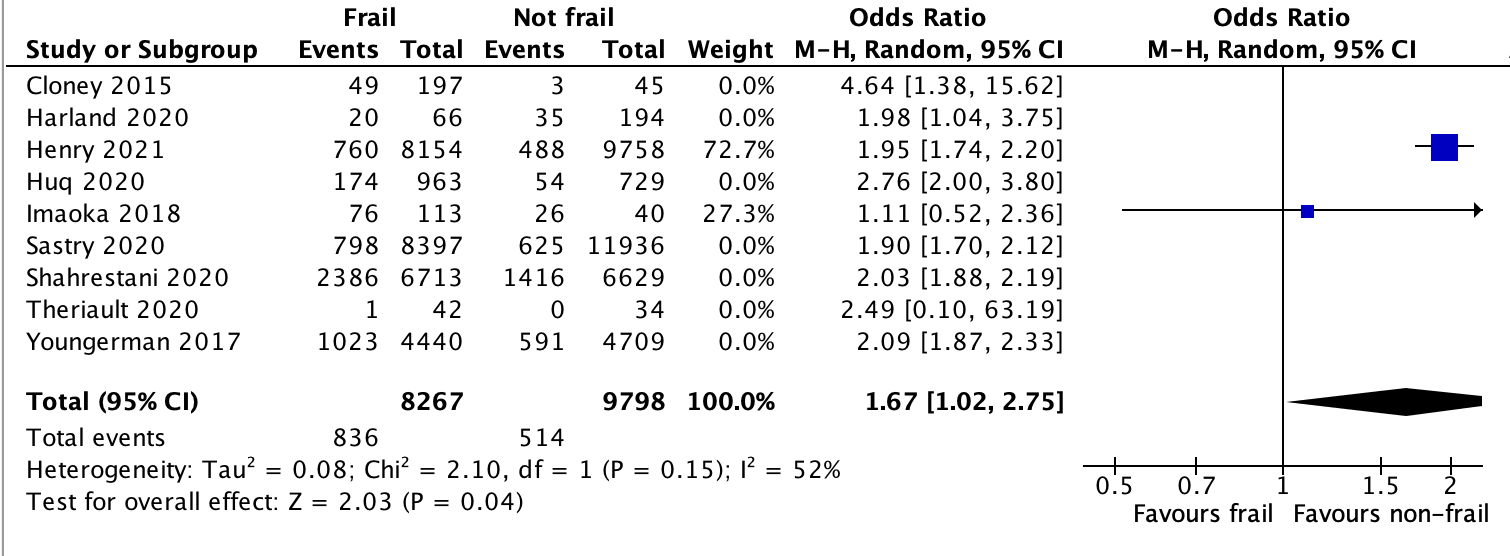
***

***Post-hoc Analysis***

*Mortality in frail patients compared to non-frail patients*

***
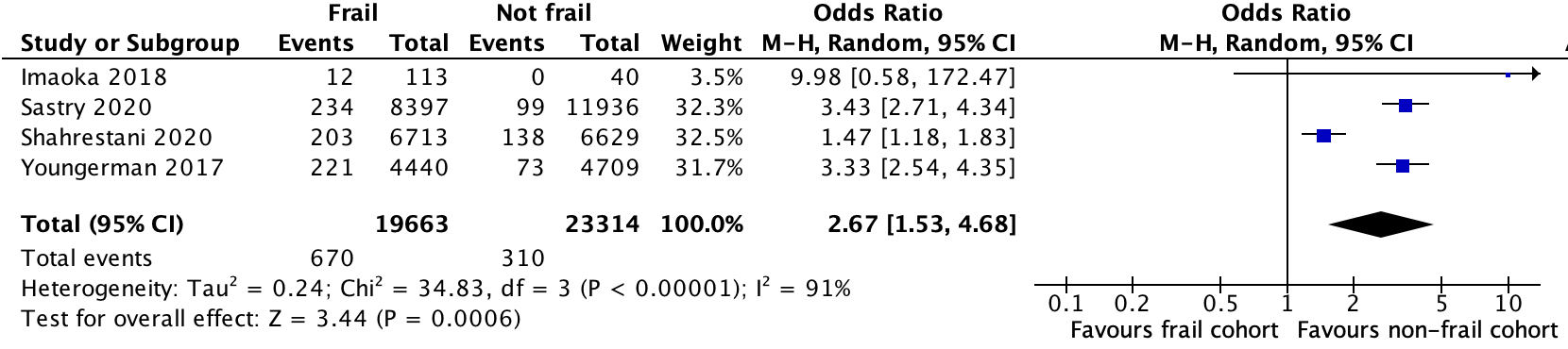
***

*Frailty using the phenotype construct only*

*
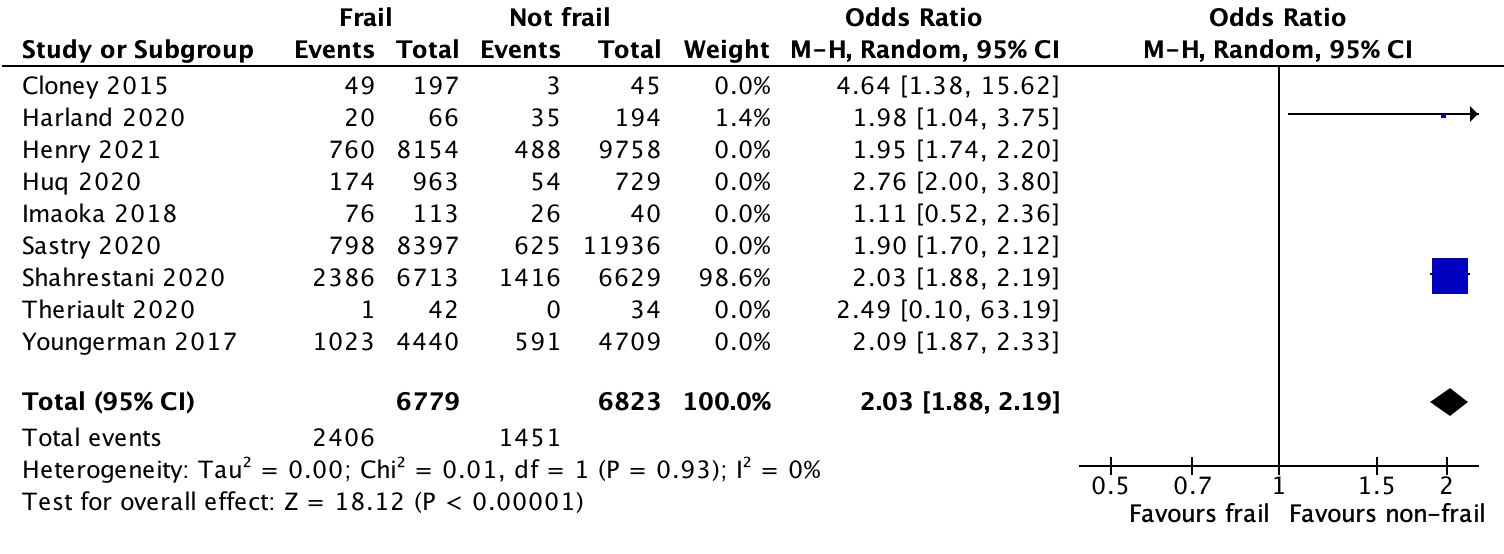
*

*Smaller (under 1000 individual patients non-database studies)*

*
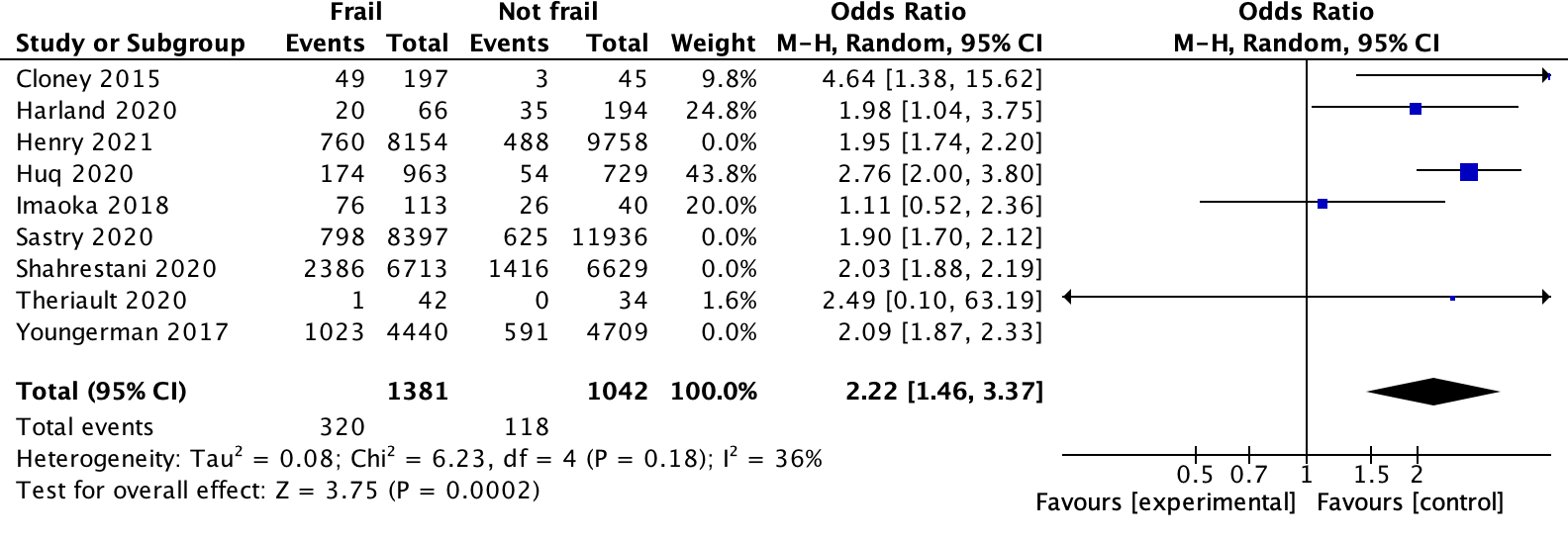
*

*Overall complications in elderly only (>65 years of age)*

***
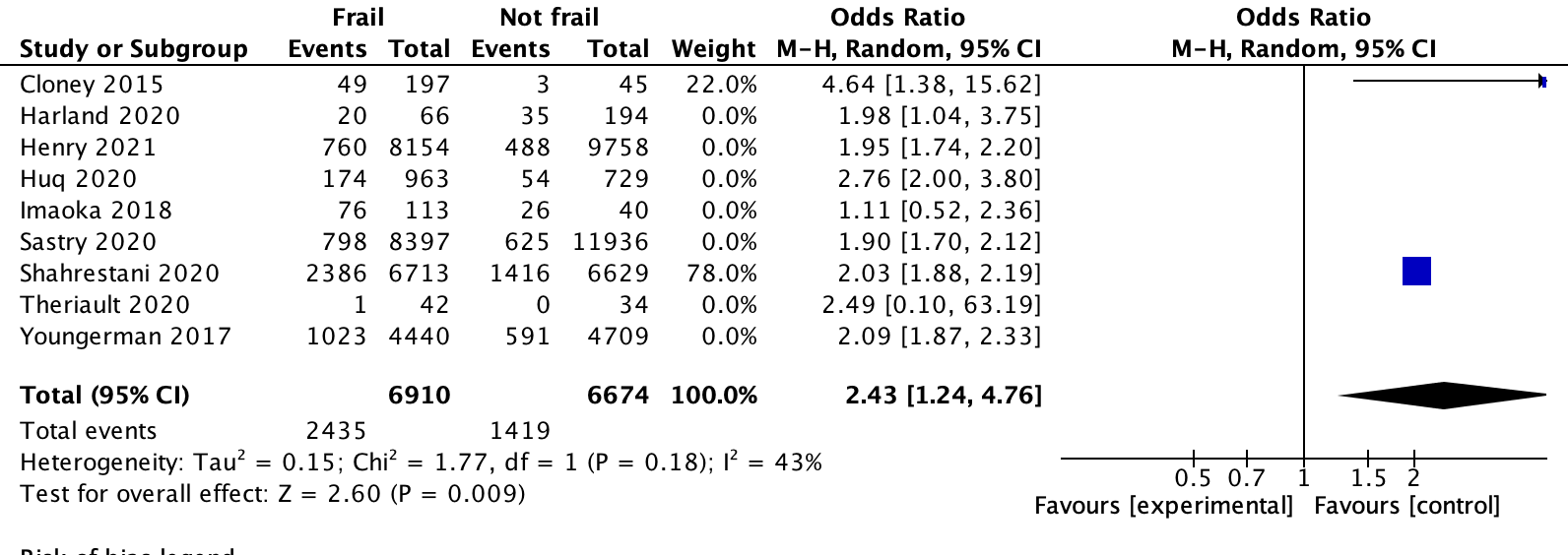
***

**Question:** Likelihood of complications and other adverse outcomes in frail patients undergoing craniotomy surgery

**Setting:** Adult patients undergoing craniotomy in a tertiary center

**Bibliography:** Nine studies eligible for this meta-analysis (1) (2-4) (5, 6) (7, 8) (9)

*Evidence Profile Tables and Summary of Findings Data*

| **Certainty assessment** | | | | | | | **№ of patients** | | **Effect** | | **Certainty** | **Importance** |
| --- | --- | --- | --- | --- | --- | --- | --- | --- | --- | --- | --- | --- |
| **№ of studies** | **Study design** | **Risk of bias** | **Inconsistency** | **Indirectness** | **Imprecision** | **Other considerations** | **frail** | **not frail** | **Relative (95% CI)** | **Absolute (95% CI)** |  |  |
| **Clavien-Dindo 1-4 Complications (follow-up: 30 days)** | | | | | | | | | | | | |
| 9 | observational studies | serious^a^ | not serious | serious | not serious | all plausible residual confounding would reduce the demonstrated effect | 5287/29085 (18.2%) | 3238/34074 (9.5%) | **OR 2.05** (1.91 to 2.20) | **82 more per 1,000** (from 72 more to 93 more) | ⨁⨁⨁◯ Moderate | Important |
| **Length of Hospital Stay (follow-up: 30 days)** | | | | | | | | | | | | |
| 5 | observational studies | serious | very serious | serious | not serious | all plausible residual confounding would reduce the demonstrated effect | 8225 | 7631 | - | SMD **0.75 SD higher** (0.49 higher to 1.01 higher) | ⨁◯◯◯ Very low | Not important |
| **Discharge to a location other than home** | | | | | | | | | | | | |
| 5 | observational studies | serious | serious | serious | not serious | all plausible residual confounding would reduce the demonstrated effect | 7614/19658 (38.7%) | 5686/23502 (24.2%) | **OR 2.16** (1.77 to 2.64) | **166 more per 1,000** (from 119 more to 215 more) | ⨁⨁◯◯ Low | Important |
| **Mortality** | | | | | | | | | | | | |
| 4 | observational studies | serious | very serious | serious | serious | all plausible residual confounding would reduce the demonstrated effect | 670/19663 (3.4%) | 310/23314 (1.3%) | **OR 2.43** (1.11 to 5.28) | **18 more per 1,000** (from 1 more to 53 more) | ⨁◯◯◯ Very low | Not important |
| **Hospital readmission in frail compared to non-frail patients** | | | | | | | | | | | | |
| 3 | observational studies | serious | very serious | serious | serious | all plausible residual confounding would reduce the demonstrated effect | 1756/15494 (11.3%) | 1825/18757 (9.7%) | **OR 1.25** (0.81 to 1.93) | **21 more per 1,000** (from 17 fewer to 75 more) | ⨁◯◯◯ Very low | Not important |

**CI:** confidence interval; **OR:** odds ratio; **SMD:** standardised mean difference

#### Explanations

a. Studies were observational, most of them were prospective and as such scored as having some concerns with regards to bias. Several studies exhibited a high concern due to missing data.

| **Summary of findings:** | | | | | | |
| --- | --- | --- | --- | --- | --- | --- |
| **Question:** Likelihood of complications and other adverse outcomes in frail patients undergoing craniotomy surgery  **Setting:** Adult patients undergoing craniotomy in a tertiary center | | | | | | |
| Outcomes | **Anticipated absolute effects^*^** (95% CI) | | Relative effect (95% CI) | № of participants (studies) | Certainty of the evidence (GRADE) | Comments |
|  | **Risk with [comparison]** | **Risk with [intervention]** |  |  |  |  |
| Clavien-Dindo 1-4 Complications (Perioperative Complications) follow-up: 30 days | 95 per 1,000 | **177 per 1,000** (167 to 188) | **OR 2.05** (1.91 to 2.20) | 63159 (9 observational studies) | ⨁⨁⨁◯ Moderate^a^ | Consistently moderate evidence with low heterogeneity. |
| Length of Hospital Stay follow-up: 30 days | - | SMD **0.75 SD higher** (0.49 higher to 1.01 higher) | - | 15856 (5 observational studies) | ⨁◯◯◯ Very low | Low level of evidence |
| Discharge to a location other than home | 242 per 1,000 | **408 per 1,000** (361 to 457) | **OR 2.16** (1.77 to 2.64) | 43160 (5 observational studies) | ⨁⨁◯◯ Low | Low level of evidence |
| Mortality | 13 per 1,000 | **32 per 1,000** (15 to 66) | **OR 2.43** (1.11 to 5.28) | 42977 (4 observational studies) | ⨁◯◯◯ Very low | Very low evidence |
| Hospital readmission in frail compared to non-frail patients | 97 per 1,000 | **119 per 1,000** (80 to 172) | **OR 1.25** (0.81 to 1.93) | 34251 (3 observational studies) | ⨁◯◯◯ Very low | No appreciable difference between the two groups with low evidence. |
| ***The risk in the intervention group** (and its 95% confidence interval) is based on the assumed risk in the comparison group and the **relative effect** of the intervention (and its 95% CI).  **CI:** confidence interval; **OR:** odds ratio; **SMD:** standardised mean difference | | | | | | |
| **GRADE Working Group grades of evidence** **High certainty:** we are very confident that the true effect lies close to that of the estimate of the effect. **Moderate certainty:** we are moderately confident in the effect estimate: the true effect is likely to be close to the estimate of the effect, but there is a possibility that it is substantially different. **Low certainty:** our confidence in the effect estimate is limited: the true effect may be substantially different from the estimate of the effect. **Very low certainty:** we have very little confidence in the effect estimate: the true effect is likely to be substantially different from the estimate of effect. | | | | | | |

#### Explanations

a. Studies were observational, most of them were prospective and as such scored as having some concerns with regards to bias. Several studies exhibited a high concern due to missing data.

1. Cloney M, D'Amico R, Lebovic J, Nazarian M, Zacharia BE, Sisti MB, et al. Frailty in Geriatric Glioblastoma Patients: A Predictor of Operative Morbidity and Outcome. World Neurosurg. 2016;89:362-7.

2. Harland TA, Wang M, Gunaydin D, Fringuello A, Freeman J, Hosokawa PW, et al. Frailty as a Predictor of Neurosurgical Outcomes in Brain Tumor Patients. World Neurosurg. 2020;133:e813-e8.

3. Imaoka Y, Kawano T, Hashiguchi A, Fujimoto K, Yamamoto K, Nishi T, et al. Modified frailty index predicts postoperative outcomes of spontaneous intracerebral hemorrhage. Clinical Neurology and Neurosurgery. 2018;175.

4. Shahrestani S, Lehrich BM, Tafreshi AR, Brown NJ, Lien BV, Ransom S, et al. The role of frailty in geriatric cranial neurosurgery for primary central nervous system neoplasms. Neurosurg Focus. 2020;49(4):E15.

5. Huq S, Khalafallah AM, Jimenez AE, Gami A, Lam S, Ruiz-Cardozo MA, et al. Predicting Postoperative Outcomes in Brain Tumor Patients With a 5-Factor Modified Frailty Index. Neurosurgery. 2020;88(1):147-54.

6. Henry RK, Reeves RA, Wackym PA, Ahmed OH, Hanft SJ, Kwong KM. Frailty as a Predictor of Postoperative Complications Following Skull Base Surgery. Laryngoscope. 2021;131(9):1977-84.

7. Theriault BC, Pazniokas J, Adkoli AS, Cho EK, Rao N, Schmidt M, et al. Frailty predicts worse outcomes after intracranial meningioma surgery irrespective of existing prognostic factors. Neurosurg Focus. 2020;49(4):E16.

8. Youngerman BE, Neugut AI, Yang J, Hershman DL, Wright JD, Bruce JN. The modified frailty index and 30-day adverse events in oncologic neurosurgery. J Neurooncol. 2018;136(1):197-206.

9. Sastry RA, Pertsch NJ, Tang O, Shao B, Toms SA, Weil RJ. Frailty and outcomes after craniotomy for brain tumor. J Clin Neurosci. 2020;81:95-100.
